# Long-Term Consumption of 6 Different Beverages and Cardiovascular Disease–Related Mortality: A Systematic Review and Meta-Analysis of Prospective Cohort Studies

**DOI:** 10.1016/j.cdnut.2024.102095

**Published:** 2024-02-08

**Authors:** Buna Bhandari, Ling Zeng, Sara Grafenauer, Aletta E Schutte, Xiaoyue Xu

**Affiliations:** 1 Central Department of Public Health, Tribhuvan University Institute of Medicine, Kathmandu, Nepal; 2 Department of Global Health and Population, Harvard T.H. Chan School of Public Health, Boston, MA, United States; 3 School of Population Health, University of New South Wales, Sydney, New South Wales, Australia; 4 School of Health Sciences, University of New South Wales, Sydney, New South Wales, Australia; 5 The George Institute for Global Health, Sydney, New South Wales, Australia; 6 Hypertension in Africa Research Team, Medical Research Council Unit for Hypertension and Cardiovascular Disease, North-West University, Potchefstroom, South Africa

**Keywords:** beverages consumption, coffee, alcohol, tea, sugar-sweetened beverages, fruit juice, energy drinks, cardiovascular mortality, long-term, meta-analysis

## Abstract

The relationship between beverage consumption and risk of cardiovascular disease has been extensively examined in cross-sectional studies. However, limited studies have investigated beverage consumption as a longer-term habitual behavior, which is important owing to potential cumulative harmful or beneficial cardiovascular effects. We examined the association between the long-term consumption of 6 types of beverages (sugar-sweetened or artificially sweetened beverages, tea, coffee, fruit juice, energy drinks, and alcohol) and cardiovascular mortality, by considering sex differences. We conducted a systematic search of MEDLINE, EMBASE, CINAHL, Web of Science, and Scopus databases from 2010 to December 2023. Of 8049 studies identified, 20 studies were included for meta-analysis. Summary hazard ratios (HRs) and 95% confidence intervals (CIs) were estimated with the use of a random-effects model. We found that long-term coffee consumption was related to reduced cardiovascular disease–related mortality in males (pooled HR: 0.63; 95% CI: 0.46, 0.87; *P* = 0.005) but not in females (HR: 0.78; 95% CI: 0.60, 1.02; *P* = 0.07). Long-term higher intake of tea was associated with lower risk of cardiovascular disease–related mortality in all adults (pooled HR: 0.81; 95% CI: 0.72, 0.92; *P* ≤ 0.001). Higher alcohol intake was linked to higher stroke in both males (pooled HR: 1.44; 95% CI: 1.06, 1.94; *P* = 0.02) and females (pooled HR: 2.26; 95% CI: 1.34, 3.81; *P* = 0.002). Higher sugar-sweetened beverage intake was in relation to higher cardiovascular disease–related mortality (pooled HR: 1.31; 95% CI: 1.16, 1.46; *P* ≤ 0.0001). We concluded that long-term habitual coffee consumption is beneficial for males, and tea consumption is beneficial for all adults. Long-term high alcohol and sugar-sweetened beverage consumption increased risk of cardiovascular disease–related mortality for both males and females. However, we were unable to draw conclusions on the potential benefit or harm of the long-term consumption of fruit juice and energy drinks on cardiovascular disease–related mortality owing to the limited number of studies available.

This review was registered at PROSPERO as CRD42020214679.

## Introduction

Cardiovascular disease (CVD) is the leading cause of death worldwide, accounting for approximately one-third of global deaths in 2019 [1]. It is well established that some risk factors, such as an unhealthy diet [1,2] and physical inactivity [3], increase risk of CVD-related mortality. The consumption of beverages [such as sugar-sweetened beverages (SSBs), tea, and coffee] is reported to be associated with cardiovascular (CV) health, particularly, CVD-related mortality. For example, a previous review [4] found that higher intake of SSBs was associated with higher CVD-related mortality, which can be patricianly explained by the presence of factors such as weight gain and type 2 diabetes. The protective role of caffeinated beverages consumption on CVD outcome was found in dose–response meta-analysis [5]. The mechanism could involve the beneficial effects of the anti-inflammatory, insulin-sensitizing, and antioxidative components in these beverages, contributing to the maintenance of glucose and cholesterol concentrations [6,7]. However, results are often inconsistent across different studies owing to different study design. This has led to the omission of specific recommendations regarding beverage consumption in many healthy dietary recommendations and guidelines [2,8].

In the existing literature, many studies used cross-sectional designs (i.e., using 1-time point data on drinking behavior) and concluded the associations between beverage consumption and CVD outcomes. However, the findings from these studies often exhibit discrepancies, introducing a level of uncertainty to the established connections between drinking habits and CVD outcomes [4,5,9,10]. This incongruity may arise from the limitation inherent in cross-sectional designs because they only capture a snapshot of behavior at a specific moment, potentially overlooking the dynamic nature of long-term habits and their relevance to CV health [11,12]. To attain a more comprehensive understanding of the intricate relationship between beverage consumption and CVD outcomes, longitudinal studies that track changes in beverage consumption behavior over time may offer valuable insights into the cumulative effects associated with CV health. In addition, it is well established that food preferences can vary between males and females, resulting in sex-specific dietary risks of CVD that diverge [13]. Although it is common that males and females often exhibit distinct patterns of beverage consumption [14,15], it remains unclear whether these differences are associated with different CVD-related mortality. Elucidating these associations in both males and females hold the potential to lay the groundwork for precise and impactful public health interventions, tailored to address the unique characteristics for both males and females.

Hence, to address gaps in the literature, this systematic review and meta-analysis was conducted to investigate the association between sex-specific long-term consumption of beverages [SSBs or artificial-sweetened beverages (ASBs), tea, coffee, fruit juices, energy drinks, and alcohol] and CVD-related mortality. We aimed to capture long-term beverage consumption behavior by including longitudinal cohort studies. Further, to comprehensively evaluate different types of beverages, we include a total of 6 types of beverages, namely alcohol, coffee, SSBs or ASBs, tea, fruit juice, and energy drinks.

## Methods

This systematic review and meta-analysis was reported in line with PRISMA statement [16]. This review was registered in PROSPERO (CRD42020214679), and first part of the review findings with separate objectives is published elsewhere [12].

### Search strategy

A comprehensive search strategy was developed by using key terms and MeSH (Medical Subject Headings) terms. Five electronic databases were targeted for searching: MEDLINE, EMBASE, CINAHL, Web of Science, and Scopus, published since 2010 and last searched in November 2022 and search was updated in December 2023 (Supplemental Table 1). Endnote X9 was employed to manage and screen the search output. After the removal of duplicates, an initial screening of the title and abstract was conducted in Covidence by 2 independent reviewers (BB, LZ); further full-text review was done (BB and LZ) to retrieve eligible articles in accordance with inclusion and exclusion criteria. Where there was disagreement on included articles, this was discussed among 3 reviewers (BB, LZ, and XX) in the Covidence until a consensus was reached.

### Study selection

Studies included in this systematic review met the following criteria: *1*) peer-reviewed and full-text original research article; *2*) prospective cohort studies; *3*) CVD-related mortality as measurement end point; *4*) measured the exposure of beverages, including SSBs or ASBs, tea, coffee, fruit juice, energy drink, and alcohol consumption at >1 time point; *5*) adults aged older than 18 y; and *6*) written in English.

The term CVD-related mortality in this review refers to the death due to CVD, such as coronary artery disease (CAD), cerebrovascular disease, and heart failure [1]. We included articles using the International Classification of Disorders [17] or clearly defined CVD-related death. We excluded studies that measured beverage consumption only at 1 time point or failed to specify the relationship between beverage consumption and CVD-related mortality.

### Data extraction

Essential data were extracted from included article by 2 independent reviewers (BB, LZ), encompassing the following: first author name, year of publication, the country where the study was conducted, cohort study name, age at entry, sex, sample size, total outcome cases, beverage consumption assessment, outcome assessment, type of beverage, follow-up years, adjusted factors, multivariate-adjusted risk estimates [hazard ratios (HRs) and relative risks (RRs), with their corresponding 95% confidence intervals (CIs) comparing highest with lowest beverage intake category].

If a study provided >1 multivariate-adjusted model, the model with the most adjustable variables was selected for the meta-analysis. If there were separate risk estimate results for male, female, and subgroups of CVD-related mortality [e.g., CAD, ischemic heart disease (IHD), or cerebrovascular disease attributed to death], these data were extracted for running separate meta-analysis. The extracted data were tabulated (Supplemental Table 2–9). The reviewers repeatedly checked the extracted data to ensure no key findings were missed. Any disagreement on extracted data was discussed among the 3 reviewers (BB, LZ, XX) until a consensus was reached.

### Quality assessment

The Newcastle–Ottawa Quality Assessment Scale [18]) was applied to evaluate the quality of included cohort studies. It consists of 3 domains to assess risk of bias: selection of the study groups (4 questions), the comparability of the groups on controlling confounders (1 question), and the measurement of outcomes (3 questions). In this review, the follow-up time to mortality occurred 10 years or more was deemed adequate. Detailed scoring criteria was explained in the tool [18], and the study achieved over 6 points and was marked as good quality. The detailed scores by 2 independent reviewers (BB and LZ) were calculated and presented in Supplementary Table 10. If there were discrepancies, this was discussed and agreed.

### Statistical analyses

A meta-analysis using a random-effect model was conducted to combine the results of different studies (HRs, RRs, or ORs), examining the relationship between different types of beverage consumption and overall CVD-, stroke-, CAD-, and IHD-related mortality. Comparisons were made between the highest category of beverage intake and the lowest category of beverage intake. The degree of variation among the cohorts was assessed using the *I*^2^ statistic, with *I*^2^ > 50% indicating significant heterogeneity. Funnel plots were used to explore the potential small-study effects such as publication bias. Owing to the small number of studies (<10) included in each beverage group meta-analysis, an Egger test was not performed, as recommended by the Cochrane Handbook [19]. The meta-analysis was performed using the RevMan software (version 5.4, The Cochrane Collaboration).

## Results

**FIGURE 1:**
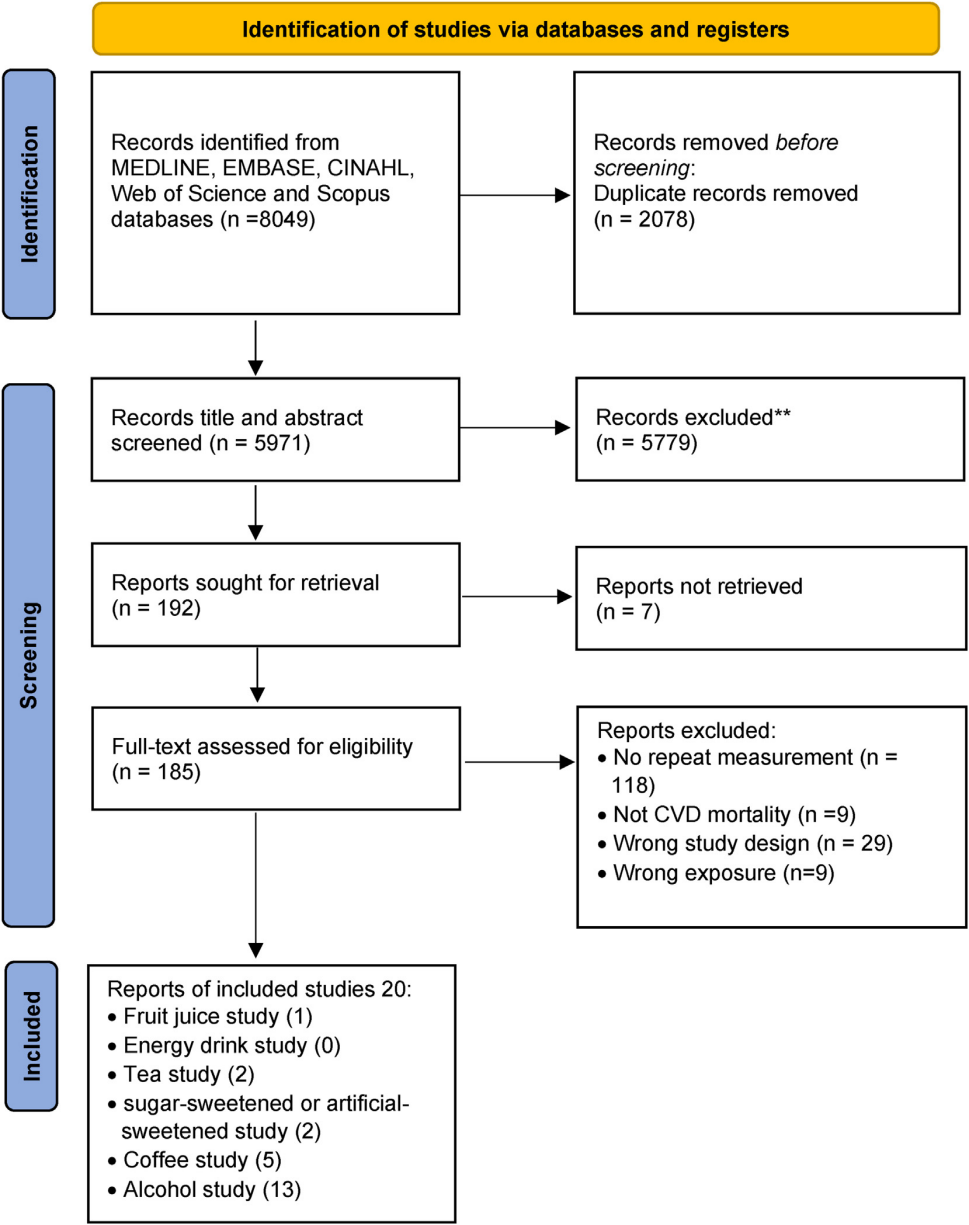
PRISMA chart.

Of the 8049 records that were identified from the literature search, 185 full-text articles were assessed in detail because they reported CVD-related mortality and different beverages consumption in the title or abstract (Figure 1). After a full-text review, a total of 20 studies were included for data extraction based on the review eligibility criteria. Among the included studies, 1 study reported on fruit juice [20], 2 studies [20,21] on tea, 2 studies [10,20] on SSBs or ASBs, 5 studies [20,[22], [23], [24], [25]] on coffee, and 13 [[26], [27], [28], [29], [30], [31], [32], [33], 34], [35], [36], [37], [38]] studies on alcohol consumption. No studies were retrieved for energy drinks.

### Characteristics of included studies

Seven studies [10,20,[23], [24], [25],^27^,^33^] were conducted in the United States, 2 in China [21,36], and each 1 in India [37], Japan [32], Croatia [22], United Kingdom [26], Serbia [38], Korea [29], Sweden [30], Netherlands [28], Europe [31], Thailand [34], and Cuba [35]. The length of follow-up ranged from 5.5 years to 40 years. Only 5 studies [22,24,[31], [32], [33]] reported sex-specific results, and 1 study [30] included only female participants, and 6 studies [[25], [26], [27], [28], [29],^37^] included only male participants. The characteristics of the included studies for meta-analysis are summarized in Supplemental Table 2–9.

In terms of methods for the dietary data collection, of a total of 20 studies, 4 studies [10,20,23,27] used a self-administered food frequency questionnaire, whereas other studies employed self-designed questionnaires. The common frequency of the assessment of beverage intake ranged from 2 to 8 times with intervals of 1–20 years. Participants were required to recall their beverage consumption over the previous year, which was the common method to assess the dietary pattern (Supplementary Table 2–9). Except for 1 study [38], all studies were assessed with fair or good quality based on the Newcastle–Ottawa risk-of-bias assessment studies (Supplementary Table 10).

### Coffee and overall CV mortality

Two studies [20,23] evaluated coffee consumption in relation to CVD-related mortality, encompassing all adults without sex-specific data (*n* = 2474 for overall CVD-related deaths), 3 studies [22,24,25] on males (*n* = 2518 for overall CVD-related deaths), and 2 studies [22,25] on females (*n* = 2549 for overall CVD-related deaths) were included in the meta-analysis, comparing from the highest with the lowest intake of coffee.

**FIGURE 2:**
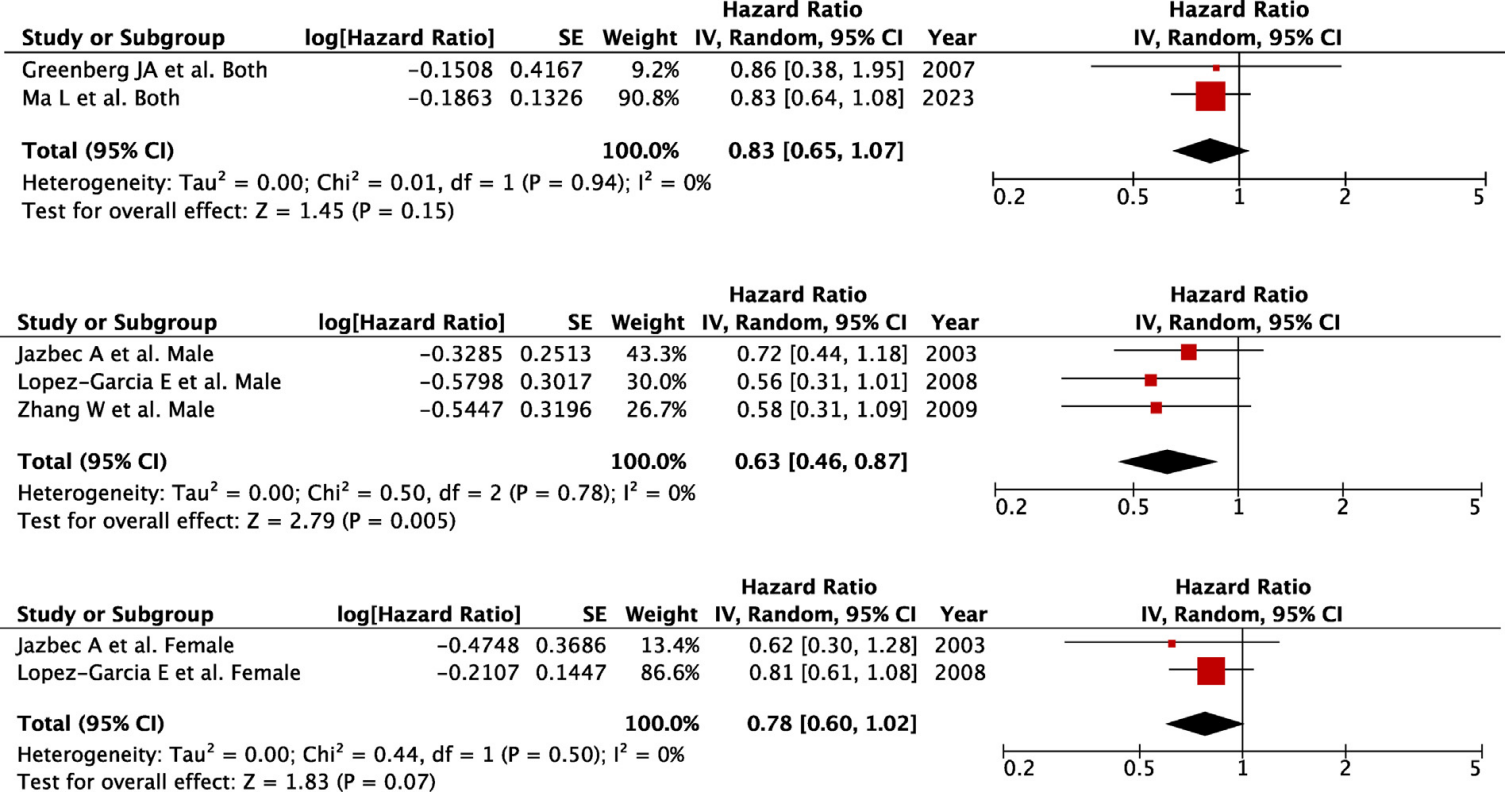
Coffee consumption and overall cardiovascular disease–related mortality in (A) both sexes, (B) males, and (C) females. Forest plots showing multivariate-adjusted hazard ratio with 95% CIs for the highest compared with the lowest coffee consumption and cardiovascular disease–related mortality. 95% CI, 95% conﬁdence interval calculated from random-effect models; IV, Inverse variation. Pooled estimates > 1 favor higher consumption and < 1 favors lower consumption.

Overall, there was no association found between coffee consumption and overall CVD-related mortality among adults with zero heterogeneity among the studies (Figure 2A) (pooled HR: 0.83; 95% CI: 0.65, 1.07; *P* = 0.15; *I*^2^ = 0%; *P*-heterogeneity = 0.50).

In males, coffee consumption was found to be protective for overall CVD-related mortality (Figure 2B) (pooled HR: 0.63; 95% CI: 0.46, 0.87; *P* = 0.005; *I*^2^ = 0%; *P*-heterogeneity = 0.78) with zero heterogeneity among the studies. However, we did not find any relationship between coffee consumption and overall CV mortality in females with zero heterogeneity among the studies (Figure 2C) (pooled HR: 0.78; 95% CI: 0.60, 1.02; *P* = 0.07; *I*^2^ = 0%; *P*-heterogeneity = 0.50). There was no severe asymmetry observed from the visual inspection of the funnel plots (Supplemental Figures 1–3).

### Alcohol and overall CVD-related mortality

**FIGURE 3:**
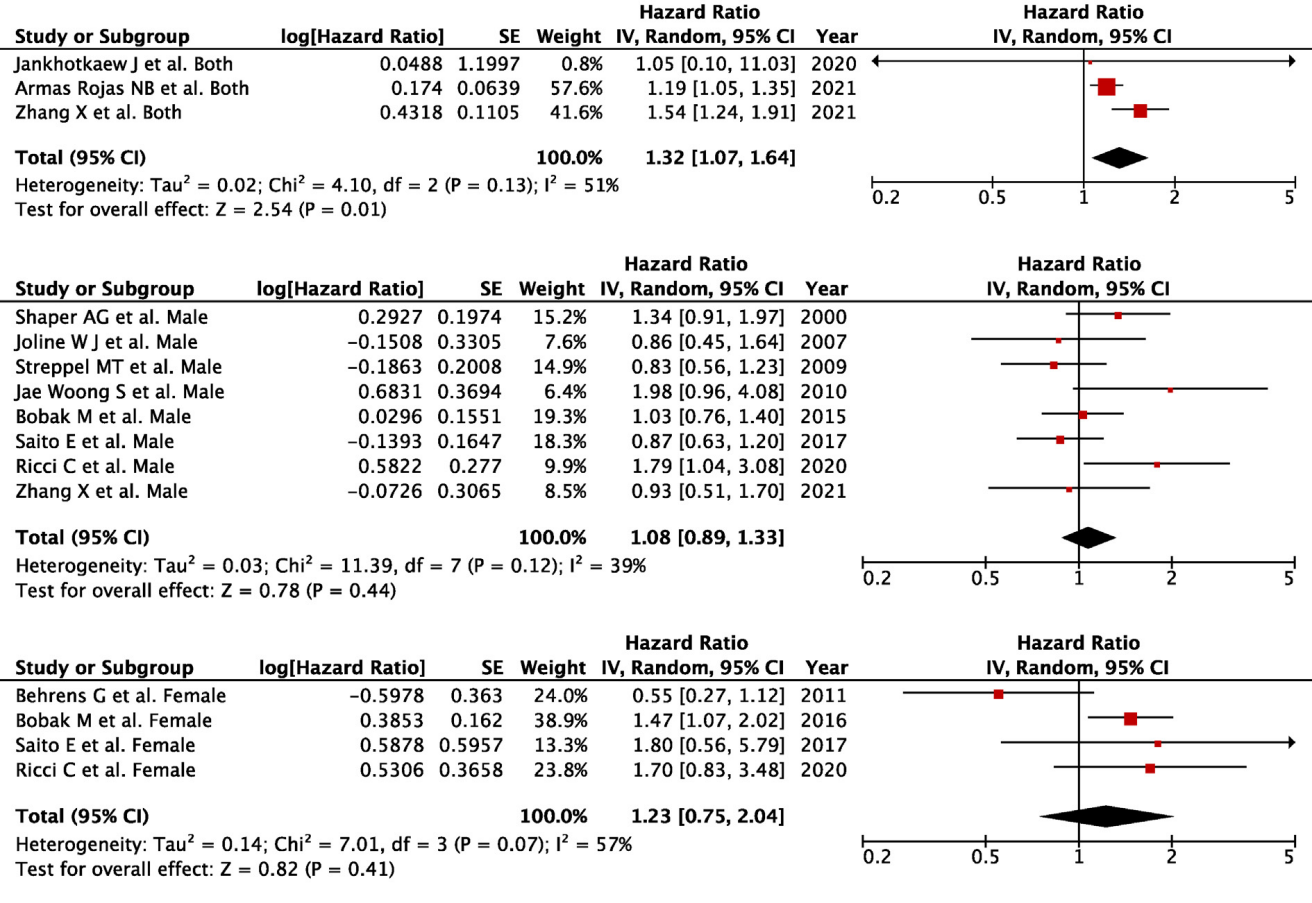
Alcohol consumption and overall cardiovascular disease–related mortality in (A) both sexes, (B) males, and (C) females. Forest plots showing multivariate-adjusted hazard ratio with 95% CIs for the highest compared with the lowest alcohol consumption and cardiovascular disease–related mortality. 95% CI, 95% conﬁdence interval calculated from random-effect models; IV, Inverse variation. Pooled estimates > 1 favor higher consumption and < 1 favors lower consumption.

Three studies [34], [35], [36]] reported data that encompassed both males and females (*n* = 6861 for CVD-related deaths) and were included in the meta-analysis comparing the highest with the lowest (Figure 3A). Higher alcohol intake was associated with a higher overall CVD-related mortality (the highest intake compared with the lowest intake; pooled HR: 1.32; 95% CI: 1.07, 1.64; *P* = 0.01; *I*^2^ = 51%; *P*-heterogeneity = 0.13).

**FIGURE 4:**
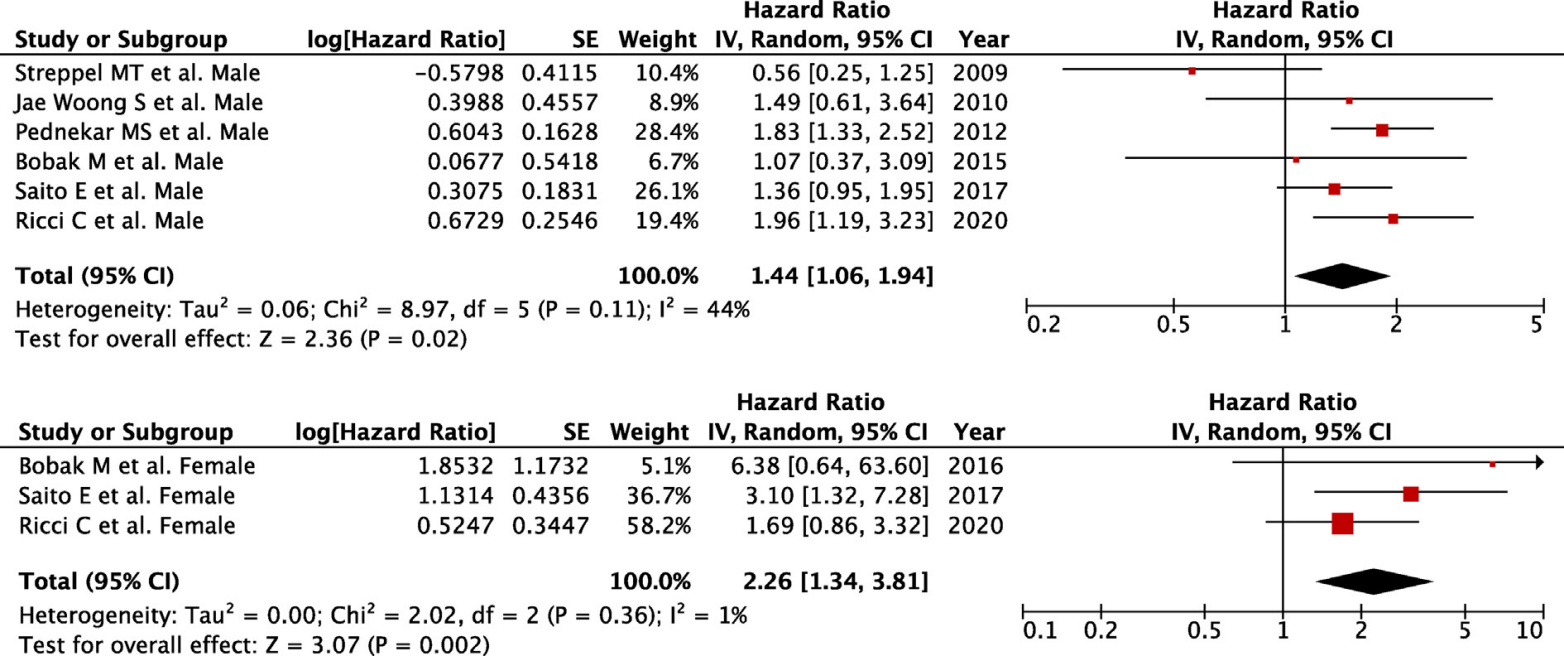
Alcohol consumption and stroke-related mortality in (A) males and (B) females. Forest plots showing multivariate-adjusted hazard ratio with 95% CIS for the highest compared with the lowest alcohol consumption and stroke mortality. 95% CI, 95% conﬁdence interval calculated from random-effect models; IV, Inverse variation. Pooled estimates > 1 favor higher consumption and < 1 favors lower consumption.

A total of 8 studies [[26], [27], [28], [29],[31], [32], [33],^36^] had sex-specific data for males (*n* = 3962 for CVD-related deaths) (Figure 3B) and 4 for females [[30], [31], [32], [33]] (*n* = 1505 for CVD-related deaths) (Figure 3C). However, no effects were found for males (pooled HR: 1.08; 95% CI: 0.89, 1.33; *P* = 0.44; *I*^2^ = 39%; *P*-heterogeneity = 0.12) or females (pooled HR: 1.23; 95% CI: 0.75, 2.04; *P* = 0.41; *I*^2^ = 57%; *P*-heterogeneity = 0.07), with no heterogeneity among the studies. There was no severe asymmetry observed from the visual inspection of the funnel plot (Supplemental Figures 4–6).

### Alcohol- and stroke-related mortality

One study [35] did not report sex-specific data, although it encompassed both males and females, leading to the conclusion that no association was found between alcohol-related and stroke-related mortality, the highest with the lowest RR was 1.13 (0.86–1.46) compared with 0.78 (0.71–0.86).

Six studies [28,29,[31], [32], [33],^37^] reported sex-specific data for males (*n* = 1969 for stroke-related deaths), and 3 [[31], [32], [33]] reported for females (*n* = 969 for stroke-related deaths) were included in the meta-analysis, comparing the highest with the lowest intake of alcohol. Higher risk of stroke-related mortality was found in males (Figure 4A) (pooled HR: 1.44; 95% CI: 1.06, 1.94; *P* = 0.02; *I*^2^ = 44%; *P*-heterogeneity = 0.06) and females (Figure 4B) (pooled HR: 2.26; 95% CI: 1.34, 3.81; *P* = 0.002; *I*^2^ = 1%; *P*-heterogeneity = 0.00), whereas comparing the extreme categories (the highest intake compared with the lowest intake) of alcohol intake with low heterogeneity between the included studies. There was no severe asymmetry observed from the visual inspection of the funnel plot (Supplemental Figures 7 and 8).

### Alcohol- and CAD-related mortality

**FIGURE 5:**
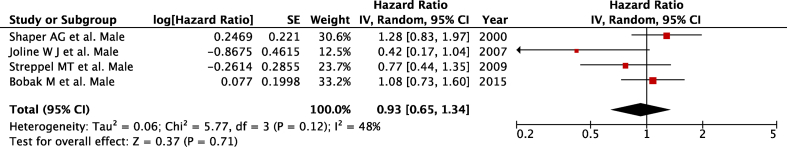
Alcohol consumption and coronary artery disease–related mortality in males. Forest plots showing multivariate-adjusted hazard ratio with 95% CIs for the highest compared with the lowest alcohol consumption and coronary artery disease mortality. 95% CI, 95% conﬁdence interval calculated from random-effect models; IV, Inverse variation. Pooled estimates > 1 favor higher consumption and < 1 favors lower consumption.

A total of 4 studies [[26], [27], [28],^31^] with a total of 1331 CAD-related mortality cases for males were included in the meta-analysis, comparing the highest with lowest intake of alcohol, but no association was found (Figure 5) (pooled HR: 0.93; 95% CI: 0.65, 1.34; *P* = 0.71; *I*^2^ = 48%; *P*-heterogeneity = 0.06). There was no severe asymmetry observed from the visual inspection of the funnel plot (Supplemental Figure 9).

### Alcohol- and IHD-related mortality

**FIGURE 6:**
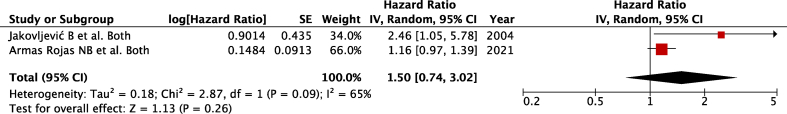
Alcohol consumption and ischemic heart disease–related mortality in both sexes. Forest plots showing multivariate-adjusted hazard ratio with 95% CIs for the highest compared with the lowest alcohol consumption and ischemic heart disease. 95% CI, 95% conﬁdence interval calculated from random-effect models; IV, Inverse variation. Pooled estimates > 1 favor higher consumption and < 1 favors lower consumption.

Two studies [35,38] encompassed both males and females, with a total of 2590 IHD-related mortality cases were included in the meta-analysis, comparing the highest with the lowest intake of alcohol. We did not find any effect of alcohol on IHD-related mortality while comparing the extreme categories (highest intake compared with lowest intake) (Figure 6) (pooled HR: 1.50; 95% CI: 0.74, 3.02; *P* = 0.26; *I*^2^ = 65%; *P*-heterogeneity = 0.18) with high heterogeneity. There was no severe asymmetry observed from the visual inspection of the funnel plot (Supplemental Figure 10).

One study [37] reported sex-specific data for males, leading to the conclusion that no association was found between long-term alcohol consumption and IHD-related mortality, comparing highest with lowest alcohol intake (HR: 0.90; 95% CI: 0.75, 1.09).

### Tea consumption and CVD-related mortality

**FIGURE 7:**
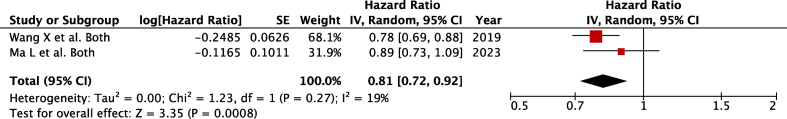
Tea consumption and overall cardiovascular disease–related mortality in both sexes. Forest plots showing multivariate-adjusted hazard ratio with 95% CIs for the highest compared with the lowest tea consumption and cardiovascular disease–related mortality. 95% CI, 95% conﬁdence interval calculated from random-effect models; IV, Inverse variation. Pooled estimates > 1 favor higher consumption and < 1 favors lower consumption.

Two studies [20,21] reported the association between long-term tea consumption and CVD-related mortality in adults, with 3874 CVD-related mortality cases in 113,673 participants. In meta-analysis, higher tea drinkers had lower risk of CVD-related mortality compared with low tea drinkers (Figure 7) (pooled HR: 0.81; 95% CI: 0.72,.92; *P* ≤ 0.001; *I*^2^ = 19%; *P*-heterogeneity = 0.027). There was no severe asymmetry observed from the visual inspection of the funnel plot (Supplemental Figure 11).

### SSBs or ASBs and CVD-related mortality

Two studies [10,20] examined the relationship between long-term SSBs (SSB) or ASB consumption in relation to CVD-related mortality in 131,134 adults, with a total of 10,293 CVD-related mortality cases included in the meta-analysis comparing the highest with the lowest beverage intake.

**FIGURE 8:**
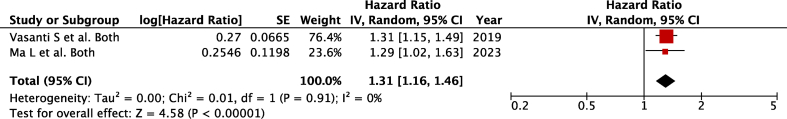
Sugar-sweetened beverage consumption and overall cardiovascular disease–related mortality in both sexes. Forest plots showing multivariate-adjusted hazard ratio with 95% CIs for the highest compared with the lowest sugar-sweetened beverage consumption and cardiovascular disease–related mortality. 95% CI, 95% conﬁdence interval calculated from random-effect models; IV, Inverse variation. Pooled estimates > 1 favor higher consumption and < 1 favors lower consumption.

**FIGURE 9:**
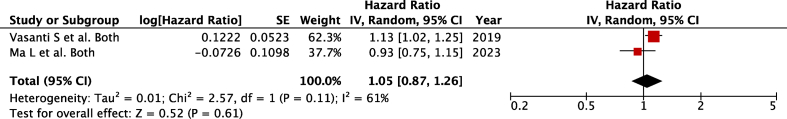
Artificially sweetened beverage consumption and overall cardiovascular disease–related mortality in both sexes. *Forest plots showing multivariate-adjusted hazard ratio with 95% CIs for the highest compared with the lowest artificially sweetened beverage consumption and cardiovascular mortality. 95% CI, 95% conﬁdence interval calculated from random-effect models; IV, Inverse variation. Pooled estimates > 1* favor higher consumption and < 1 favors lower consumption.

Higher SSB intake was associated with a higher overall CVD-related mortality (the highest intake compared with the lowest intake) (Figure 8) (pooled HR: 1.31; 95% CI: 1.16, 1.46; *P* ≤ 0.0001; *I*^2^ = 0%; *P*-heterogeneity = 0.91). However, we did not find any effect of ASB on CVD-related mortality while comparing the extreme categories (highest intake compared with lowest intake) (Figure 9) (pooled HR: 1.05; 95% CI: 0.87, 1.26; *P* = 0.61; *I*^2^ = 61%; *P*-heterogeneity = 0.11) with high heterogeneity. There was no severe asymmetry observed from the visual inspection of the funnel plot (Supplemental Figures 12 and 13).

### Fruit juice consumption and CVD-related mortality

Only 1 study [20] reported the association between long-term fruit juice consumption and CVD-related mortality, with 2397 CVD-related mortality cases in 12,771 participants. There was no significant relationship found between fruit juice consumption and CVD-related mortality (HR: 1.07, 95% CI: 0.92, 1.23; *P* = 0.66), comparing the highest intake (>1 serving per day) with lowest intake (<1 serving per month).

## Discussion

This review analyzed the findings from 20 prospective cohort studies that investigated the association between a long-term intake of various beverages and CVD-related mortality. The majority of literature reporting health benefits and harms of drinks, in particular alcohol consumption, have focused on 1-time reporting of intake. We found that long-term coffee consumption was associated with a lower CVD-related mortality but only in males. High alcohol consumption in the long-term was related to higher risk of overall CVD-related mortality in adults but no relationships were found in sex-specific groups. Long-term consumption of alcohol increased risk of stroke-related mortality in both males and females but was associated with neither CAD-related nor IHD-related mortality. Long-term higher intake of tea was associated with lower risk of overall CVD-related mortality whereas the higher SSB intake with higher CVD-related mortality in both sexes. No data allowed us to perform meta-analysis on fruit juice and energy drinks in relation to CVD-related mortality.

We found that males with the highest coffee consumption (>2–6 cups per day) had lower risk of CVD-related mortality by 37%. No association was found in females and both sexes, which can be attributed to the small number of articles included in our meta-analysis. Limited review studies explored the associations between long-term coffee consumption and CVD-related mortality, with most studies primarily focused on assessing the cross-sectional association between coffee consumption and CVD risk or mortality [39]. For example, a recent review [40] of including 12 prospective cohorts (*n* = 248,050), only measuring the coffee consumption at baseline concluded an inverse association between coffee consumption (≥5 cups per day) and CVD-related mortality in males but not in females. Thus, further research is encouraged to examine the relationship between long-term coffee consumption and CVD-related mortality.

Our study indicated that high, long-term alcohol consumption was associated with an increased overall CVD-related mortality by 32%. Regardless of whether alcohol consumption was short-term or long-term, some review articles [41,42] have illustrated a U- or J-shaped association between alcohol consumption and CVD-related risk or mortality in both sexes and only males. With the evidence of the detrimental impact of excessive alcohol intake on CVD outcomes becoming more pronounced over the past 2 decades [43]. Interestingly, when we looked at sex-specific analysis, no effects were observed. Thus, further studies in exploring the sex-specific long-term effects of alcohol consumption and overall CVD-related mortality are needed.

Long-term alcohol consumption was associated with higher risk of stroke mortality in sex-specific analyses. Similarly, limited studies have examined the link between long-term alcohol consumption and stroke-related mortality, and some review findings regarding short-term alcohol consumption and its impact on stroke incidence or related mortality may now be outdated and inconclusive [44,45]. Using available data on CAD in males and IHD in both sexes, our findings indicate no association between heavy alcohol consumption and CAD-related or IHD-related mortality.

Very limited studies have been performed to examine long-term tea and SSB or ASB in relation to CVD-related mortality because we found only 2 studies for tea and SSB or ASB intake, respectively, for meta-analysis, higher tea consumption was related to a 19% decrease in CVD-related mortality, whereas higher SSB with 31% increased risk of CVD-related mortality in both sexes. These results are echoed with studies examined the short-term cross-sectional link between tea consumption and CVD-related mortality, indicating that each additional daily cup (236.6 mL) increase in tea consumption was related to a 4% reduction in risk of CVD-related mortality [9]. Each additional daily serving of SSB (equivalent to 355 mL) has been shown to increase CVD-related mortality by 8% among 896,005 participants [4]. However, our results were based on the longitudinal studies that tracked changes in tea and SSB consumption behavior over time, which could offer valuable insights into the cumulative effects associated with cardiovascular health.

Previous studies have reported that a higher fruit juice intake was associated with a higher CVD-related mortality by 32% in 3,013,817 participants [46], and energy drinks may be associated with cardiac arrhythmias, cardiac arrest, and myocardial ischemia [47]. However, not enough studies were retrieved to meta-analyze the long-term intake of fruit juice and energy drinks in relation to CVD-related mortality, indicating further studies are needed.

### Strengths and limitation

To our knowledge, this is the first systematic review to evaluate the long-term effects of different types of beverages on CVD-related mortality by only including studies that had repeated measures of beverage consumption. However, our results must be interpreted cautiously. First, most studies included in this review employed self-reported questionnaires to measure beverage consumption, which may generate measurement bias. Second, in line with our inclusion criteria, there were limited articles evaluating the association between long-term consumption of fruit juice, SSB, tea, and energy drink with CVD-related mortality that allowed us to conduct meta-analysis. Finally, although we aimed to perform sex-specific analysis with specific types of CVD-related mortality (e.g., CAD-related and IHD-related mortality), there were limited studies retrieved for further analysis.

### Conclusions

Although a major proportion of the studies evaluated beverage intake and CVD-related mortality, the studies used 1 data point for dietary analyses, and thus limited studies have reviewed long-term beverage intake in relation to CVD-related mortality. Our meta-analysis found that long-term habitual coffee consumption is beneficial for males. High alcohol consumption over long-term increased risk of CVD-related mortality among all adults but not evidenced by sex-specific analysis. High alcohol consumption over long-term increased risk of stroke mortality for males and females but neither was associated with CAD nor IHD. Drinking tea in a long-term was related to lower risk of CVD-related mortality whereas high SSB drinking habit in relevance to increased risk of CVD-related mortality among general population. We were unable to draw conclusions regarding the associations between long-term consumption of fruit juice and energy drinks on CVD-related mortality owing to limited studies that have performed the analysis. Therefore, we encourage further studies in the field including sex-specific analysis to help provide evidence for beverages in dietary guidance.

## Author contributions

The authors’ responsibilities were as follows – BB, LZ, XX: protocol development and searches; BB, LZ, XX: screening; BB, LZ, XX: full-text review and data extraction, risk-of-bias assessment and synthesis; BB, LZ, XX: meta-analysis; BB, LZ, XX: wrote the first draft of the manuscript; and all authors: contributed to the research design, reviewed and commented on versions of the manuscript, and read and approved the final manuscript.

## Conflict of interest

The authors report no conflicts of interest.

## Funding

The authors reported no funding received for this study.

## Appendix A Supplementary data

Multimedia component1 Multimedia component1
