# Supplementary material for: Long-Term Consumption of 6 Different Beverages and Cardiovascular Disease–Related Mortality: A Systematic Review and Meta-Analysis of Prospective Cohort Studies
Source: Curr Dev Nutr. 2024 Feb 8;8(3):102095. doi: 10.1016/j.cdnut.2024.102095 (PMC10904171; doi:10.1016/j.cdnut.2024.102095)
Supplement: Multimedia component1 [file mmc1.docx]

**Supplementary Table 1. Search strategy**

| **Databases** | **Concept 1**  Juice, Alcohol and beverages  Sugar-sweetened beverages, Coffee, Tea, Energy drinks | **Concept 2**  Cardiovascular disease mortality | **Concept 3**  Prospective, cohort, longitudinal study |
| --- | --- | --- | --- |
| **Medline** | "juices"/ or fruit juice.mp. or fruit juice*.mp. or Alcohols/ or alcohol.mp. or Alcoholic Beverages/ or Beverages/ or Beverages.mp. or Sugar-Sweetened Beverages/ or Beverages/ or sugar-sweetened beverages.mp. or Carbonated Beverages/ or Artificially Sweetened Beverages/ or Coffee/ or coffee.mp. or Tea/ or Tea.mp. or Energy Drinks/ or ("energy drink*" or "stimulant drink*").mp. | Death/ or Death.mp or Death, Sudden/ or Death, Sudden, Cardiac/OR mortality or cardiovascular mortality  **AND**  cardiovascular disease.mp or cardiovascular diseases/ or stroke/ or stroke.mp OR cerebrovascular disorders/ or cerebrovascular disease.mp OR cerebrovascular accident.mp OR cerebral infarction or cerebral haemorrhage/ or intracerebral haemorrhage.mp or subarachnoid haemorrhage/ or subarachnoid haemorrhage.mp or coronary disease/ or coronary heart disease.mp OR coronary artery disease/ or coronary artery diseae.mp OR myocardial Ischemia/ or acute coronary syndrome/ or angina pectoris/ or myocardial infraction/ or acute myocardial infarction.mp OR myocardial infarction Or acute coronary syndrome/ or acute coronary syndrome.mp OR heart failure/ or heart failure.mp OR cardiac failure.mp OR cardiac insufficiency.mp or cardiac arrest.mp or heart arrest/ or congestive heart failure.mp or heart attack.mp or Cardiovascular events.mp or Major acute cardiovascular event.mp | prospective studies/ or prospective.mp OR follow-up OR cohort analysis/ or cohort studies.mp OR longitudinal study/ or longitudinal.mp or case cohort or nested case control.mp |
| **Embase** | (Fruit and vegetable juices).mp or fruit juice.mp or fruit juice*.mp or Alcohols.mp. or alcohol derivative/ or Beverages.mp. or beverage/ or Alcoholic Beverages.mp. or alcoholic beverage/ or Carbonated Beverages.mp. or carbonated beverage/ or sugar-sweetened beverages/ or Sugar-Sweetened Beverages.mp or sugar sweetened beverages.mp or Artificially Sweetened Beverages.mp. or artificially sweetened beverage/ or coffee/ or Coffee.mp or tea/ or Tea.mp or Energy Drink/ or ("energy drink*" or "stimulant drink*").mp. | death/ or sudden death/ or Death.mp. or sudden cardiac death/ OR mortality/ or cardiovascular mortality/or mortality.mp  **AND**  cardiovascular disease.mp or cardiovascular diseases/ or stroke.mp OR cerebrovascular accident/ OR cerebrovascular disorders .mp or cerebrovascular disease/ OR cerebral infarction.mp or brain infraction/ or cerebral haemorrhage/ or brain haemorrhage/ or intracerebral haemorrhage.mp or subarachnoid haemorrhage/ or subarachnoid haemorrhage.mp or coronary disease.mp or coronary artery disease/ OR myocardial ischemia.mp or heart muscle ischemia/ or angina pectoris/ or myocardial infraction.mp or heart infraction/ or acute coronary syndrome.mp or acute coronary syndrome acute heart infraction/ or unstable angina pectoris/ OR heart failure/ or heart failure.mp OR cardiac failure.mp OR cardiac insufficiency.mp or cardiac arrest.mp or heart arrest/ or congestive heart failure.mp or congestive heart failure/ or heart attack.mp or Cardiovascular events.mp or Major acute cardiovascular event.mp | prospective studies/ or prospective.mp OR follow-up OR cohort analysis/ or cohort studies.mp OR longitudinal study/ or longitudinal.mp or case cohort or nested case control.mp |
| **CINAHL** | "Fruit and vegetable juice*" OR "Fruit juice*" OR alcohol* OR beverage* OR "Alcoholic Beverage*" OR "Carbonated Beverage*" OR "Sugar-Sweetened Beverage*" OR "Artificially Sweetened Beverage*" OR coffee* OR tea* OR "energy drink*" OR "stimulant drink*" | Death* OR “Death, Sudden*” OR “Death, Sudden, Cardiac*” OR mortality* OR “cardiovascular mortality*”  **AND**  “cardiovascular disease*” OR stroke* OR “cerebrovascular disorders*” OR “cerebrovascular disease*” OR “cerebrovascular accident*” OR “cerebral infarction*” OR “cerebral haemorrhage*” OR “intracerebral haemorrhage*” OR “subarachnoid haemorrhage*” OR “coronary disease*” or “coronary heart disease*” OR “coronary artery disease*” OR “myocardial Ischemia*” or “acute coronary syndrome*” or “angina pectoris*” or myocardial infraction*” or “acute myocardial infarction*” OR “heart failure*” OR “cardiac failure*” OR “cardiac insufficiency*” or “cardiac arrest*” or “heart arrest*” or “congestive heart failure*” or “heart attack*” or “Cardiovascular events*” or “Major acute cardiovascular event*” | “prospective studies*” OR “follow up*” OR “cohort analysis*” or “cohort studies*”OR “longitudinal study*” or longitudinal* or “case cohort*” or “nested case-control*” |
| **Web of science** | "Fruit and vegetable juice*" OR "Fruit juice*" OR alcohol* OR beverage* OR "Alcoholic Beverage*"  OR "Carbonated Beverage*"  OR "Sugar-Sweetened Beverage*" OR "Artificially Sweetened Beverage*"   OR coffee* OR tea*  OR "energy drink*" OR "stimulant drink*" | Death* OR “Death, Sudden*” OR “Death, Sudden, Cardiac*” OR mortality*OR “cardiovascular mortality*”  **AND**  “cardiovascular disease*” OR stroke* OR “cerebrovascular disorders*” OR “cerebrovascular disease*” OR “cerebrovascular accident*” OR “cerebral infarction*” OR “cerebral haemorrhage*” OR “intracerebral haemorrhage*” OR “subarachnoid haemorrhage*” OR “coronary disease*” or “coronary heart disease*” OR “coronary artery disease*” OR “myocardial Ischemia*” or “acute coronary syndrome*” or “angina pectoris*” or “myocardial infraction*” or “acute myocardial infarction*” OR “heart failure*” OR “cardiac failure*” OR “cardiac insufficiency*” or “cardiac arrest*” or “heart arrest*” or “congestive heart failure*” or “heart attack*” or “Cardiovascular events*” or “Major acute cardiovascular event*” | “prospective studies*” OR “follow up*” OR “cohort analysis*” or “cohort studies*”OR “longitudinal study*” or longitudinal* or “case cohort*” or “nested case-control*” |
| **Scopus** | TITLE-ABS-KEY ( "fruit and vegetable juice*" OR "fruit juice*" OR alcohol* OR beverage* OR "alcoholic beverage*" OR "carbonated beverage*" OR "sugar-sweetened beverage*" OR "artificially sweetened beverage*" OR coffee* OR tea* OR "energy drink*" OR "stimulant drink*") | TITLE-ABS-KEY ( death* OR "death, sudden*" OR "death, sudden, cardiac*" OR mortality* OR "cardiovascular mortality*")  AND  TITLE-ABS-KEY ( "cardiovascular disease*" OR stroke* OR "cerebrovascular disorders*" OR "cerebrovascular disease*" OR "cerebrovascular accident*" OR "cerebral infarction*" OR "cerebral haemorrhage*" OR "intracerebral haemorrhage*" OR "subarachnoid haemorrhage*" OR "coronary disease*" OR "coronary heart disease*" OR "coronary artery disease*" OR "myocardial ischemia*" OR "acute coronary syndrome*" OR "angina pectoris*" OR "myocardial infraction*" OR "acute myocardial infarction*" OR "heart failure*" OR "cardiac failure*" OR "cardiac insufficiency*" OR "cardiac arrest*" OR "heart arrest*" OR "congestive heart failure*" OR "heart attack*" OR "cardiovascular events*" OR "major acute cardiovascular event*" ) | TITLE-ABS-KEY ( "prospective studies*" OR "follow up*" OR "cohort analysis*" OR "cohort studies*" OR "longitudinal study*" OR longitudinal* OR "case cohort*" OR "nested case-control*" ) |

**Supplementary Table 2 included study characteristics of investigating the association between coffee consumption and risk of cardiovascular mortality in adults**

| Authors | Year | Country | Cohort name | Age at entry | Sex | Sample size | Total cases | Dietary assessment | Outcome assessment | Type of coffee | Follow up years | Adjusted factors | Results (The highest vs. the lowest intake category) Hazard Ratio (95% CI) |
| --- | --- | --- | --- | --- | --- | --- | --- | --- | --- | --- | --- | --- | --- |
| Jazbec A et al (20) | 2003 | Croatia | Investigation of chronic diseases in Croatia | NA | Male & Female | Male 1571  Female  1793 | Male 254 CVD deaths  Female 181 CVD deaths | Coffee related questionnaire  2 times in 10 years interval | Deaths were collected from the official death certificate records and church membership records | Coffee | 10 |  | **Male**  Regularly >2 cups/day VS Never HR:  0.721 (0.440-1.183), P 0.1956  **Female**  Regularly >2 cups/day VS Never HR:  0.622 (0.302-1.279)  P 0.1967 |
| Greenberg JA et al.(21) | 2007 | USA | National Health and Nutrition Examination Survey (NHANES I)  Epidemiologic Follow-Up Study (NHEFS) | 25–74 | Total | 4881 | 77 CVD deaths | FFQ  4 times in 1,2 5 years interval | Each death was confirmed by death certificate or  proxy interview | Caffeinated beverage (ground caffeinated coffee, instant caffeinated  coffee, regular tea. Colas) | 8.8 | Age, smoking, BMI, sex, race, physical activity, alcohol consumption, per capita, income, educational level, and American-style diet, as described in Methods. | Servings of caffeinated beverage/day > 4 VS <0.5 RR: 0.86 (0.38, 1.06) P 0.23 |
| Lopez-Garcia E et al (22) | 2008 | USA | NHS (Nurses’ Health  Study) and HPFS (Health Professionals Follow-up Study) | NA | Male & Female | Male  41736  Female 86 214 | Male 2049 CVD deaths  Female 2368 CVD deaths | Coffee related questionnaire  4 times in 4 years interval in HPFS; 6 times in 2,4 years interval in NHS.  Recalled beverage consumption over previous year | Deaths were reported by the next of kin or the postal  authorities or were ascertained through the National Death  Index. | coffee and other caffeinated beverages (tea, caffeinated soft  drinks) | 18 years in men  24 years  in women | Age (5-year categories); smoking status (never; past; and currently smoking 1 to 14, 15 to 24, and >25 cigarettes/day); body mass index <23.0, 23.0 to 24.9, 25.0 to 27.9, 28.0 to 29.9, and >30.0 kg/m2); physical activity (quintiles of metabolic equivalent tasks in h/wk for men, and 1.0, 1.0 to 1.9, 2.0 to 3.9, 4.0 to 6.9, and>7.0 h/wk for women); alcohol intake (never, 0.1 to 4.9, 5.0 to 9.9, 10.0 to 14.9, 15.0 to 29.9, and >30.0 g/d); parental history of myocardial infarction; menopausal status | **Male**  > 6/d VS <1/mo RR:  0.56 (0.31-1.03), P 0.03  **Female**  > 6/d VS<1/mo RR:  0.81 (0.61-1.06), P <0.001 |
| Zhang W et al (23) | 2009 | USA | The Health Professionals Follow-up  Study (HPFS) | 40–75 | Diabetic male | 3497 | 215 CHD or stroke deaths | Semi quantitative frequency questionnaire  5 times in 4 years interval | Deaths were reported by next of kin or the postal system or ascertained through the National Death Index | Caffeinated coffee | Between 1986 and 2004  (24,121 person-years of follow-up) | Age (5-year categories), smoking status (never, past, or current at 1–14 or >15 cigarettes/day), BMI (<23.0, 23.0 –24.9, 25.0 –29.9, or >30.0 kg/m2),  alcohol intake (0, 0.1– 4.9, 5.0 –14.9, or >15 g/day), parental history of myocardial infarction, history of hypertension, hypercholesterolemia, physical activities  (quintiles of METs/week), duration of diabetes (<5, 5–10, or >10 years), and hypoglycemic medication (yes or no),  total energy intake; multivitamin use and vitamin E supplement use; intake of polyunsaturated, saturated, and trans fat; long-chain n-3  fatty acids; cereal fiber; folate; glycemic load (all in quintiles); and decaffeinated coffee and tea consumption, except for decaffeinated coffee and tea consumption. | > 2/day VS None RR:  0.58 [0.31–1.10] , P 0.26 |
| Ma L et al | 2023 | US | Nurses’ Health Study  Health Professionals Follow-Up Study | 30yr  40yr | Females with type 2 DM  Males with type 2 DM | 9252  3519 | 2397 CVD deaths | Food frequency questionnaire  Collected at baseline and updated every two to four years | Reports by the next of kin or postal authorities or from searches of the National Death Index | Caffeinated and decaffeinated coffee | 18.5 | Analyses were adjusted for age (continuous), duration of diabetes mellitus (years), sex (men or women), white ethnicity (yes or no), physical activity (<3.0, 3.0-8.9,  9.0-17.9, 18.0-26.9, ≥27.0 metabolic equivalents-hours/week), smoking status (never, former, current 1-14 cigarettes/day, current ≥15 cigarettes/day), alcohol consumption (0, 0.1-4.9, 5.0-14.9, ≥15.0 g/day), menopausal status and post-menopausal hormone use (pre-menopause, post-menopause (never, former, or current hormone use), or missing; Nurses’ Health Study only), family history of type 2 diabetes (yes or no) or myocardial infarction (yes or no), intake of total energy (continuous), the modified Alternative Healthy Eating Index score (fourths), history of hypertension (yes or no) or hypercholesterolemia (yes or no), use of antihypertensive (yes or no) or lipid lowering drug (yes or no), aspirin use (yes or no), diabetes drug use (oral drug only, insulin use, or others), and change in body mass index before to after diabetes diagnosis. Individual beverage consumption was mutually adjusted. | CVD mortality with coffees>4 serving/day HR VS <1 serving/month HR:  0.83 (0.64 to 1.08)  P <0.001 |

**Table 3. Included study characteristics of investigating the association between Alcohol consumption and risk of cardiovascular mortality in adults**

| **Author** | **Year** | **Country** | **Cohort name** | **Age at entry** | **Sex** | **Sample size** | **Total cases** | **Dietary assessment** | **Outcome assessment** | **Type of alcohol** | **Follow up years** | **Adjust factors** | **Results (The highest vs. the lowest intake category) HR or RR (95% CI)** |
| --- | --- | --- | --- | --- | --- | --- | --- | --- | --- | --- | --- | --- | --- |
| Shaper AG et.al (24) | 2000 | UK | The British regional heart study | 45–64 | Male with CHD | 655 | 208 CVD deaths | Alcohol  Questionnaire  2 times in 5 years interval | National Health Service registers | Alcohol | 12.8 | Age, smoking, BMI, social class, pre-existing diabetes, stroke, and regular medication | Moderate/heavy VS Teetotallers RR :  1.34 (0.91 to 1.98) VS 0.98 (0.53 to 1.82) |
| Beulens JW et al (25) | 2007 | USA | Health Professionals Follow-Up Study | 40-75 | Male with hypertension | 11711 | 394 CVD  deaths | food-frequency questionnaire  4 times in 4 years interval  Recalled consumption over previous year | Medical records or autopsy reports | Beer, white wine,  red wine, and liquor | 16 | Age; smoking; body mass index; physical activity; diabetes; hypercholesterolemia; family history of MI; aspirin use; lipid-lowering therapy; energy intake; and  energy-adjusted quintiles of saturated fat, trans fatty acids, sodium, potassium, magnesium, folate, vitamin E, -3 fatty acids, and dietary fiber. | > 50g/d VS 0 HR:  0.86 (0.45–1.63)  P trend 0.35 |
| Streppel M.et al (26) | 2009 | Netherlands | Zutphen Study | Middle-aged | Male | 1373 | 628 CVD deaths | Cross-check dietary history method  7 times in 5 years interval  Recalled consumption over previous year | Official death certificate | Beer, wine, spirits | 40 | Adjusted for former drinking, energy intake without alcohol, the  number of cigarettes smoked, cigarette smoking duration, cigar or pipe smoking,  intake of vegetables, fruit, fish, saturated fat and trans fatty acids, body mass index,  prevalence of myocardial infarction, stroke, diabetes mellitus and cancer, and baseline  socioeconomic status. | > 20g/day VS No drinker HR:  0.83 (0.56 to 1.22) |
| Jae Woong S et al (27) | 2010 | Korea | A Kangwha Cohort Study | > 55 | Male | 2600 | 337 CVD deaths | Alcohol consumption questionnaire  2 times in 9 years interval | Records of burial and death  certificates of eup and myeon offices, through the family’s confirmation of cause of death, or the national data on cause of death. | Consumed most soju and makkoli. | 20.8 | Age (year of recruitment), history of chronic disease, history of diabetes, smoking habits (never, former, 1–19 tobacco/day, and >20 tobacco/day), body mass index, hypertension status, occupation (agriculture, other), and education status, total alcohol consumption, excluding subjects who had been followed up <5 years. | Heavy (>12 Drinks) VS Non-drinker HR  1.98 (0.96–4.10) |
| Behrens G et al (28) | 2011 | Sweden | Swedish women’s lifestyle and health (WLH)cohort study | 30-49 | Female | 47921 | 158 CVD deaths | a self-report questionnaire  2 times in 12 years interval  Recalled current consumption status | The national registers | Beer, wine, spirits | 16 (08/1991 -12/2006) | Age (5-year categories), BMI (\18.5, 18.5–24.9, 25.0? kg/m2), energy-adjusted saturated fat intake (quintiles), smoking status  (never, past, and current smoking with current intensities of 1–14 cigs/day, 15–24 cigs/day, and 25? cigs/day), current physical activity (low, intermediate, high), years of education (0–10, 11–13, 14? years, where 10 years of education correspond to completion of compulsory school,  13 years of education correspond to completion of secondary school, and 14 years of education or more correspond to graduate study at university) | >10g/day VS Non-drinker RR:  0.55 (0.27–1.12)  P trend: 0.26 |
| Bobak M et al (29) | 2016 | Four Central and Eastern European countries | The Health, Alcohol and Psychosocial factors In Eastern Europe (HAPIEE) s | 45-69 | Male & Female | Male 15989  Female  18315 | Male  833 CVD deaths  Female  389 CVD deaths | Graduated frequency questionnaire  More than 2 times in 3.5 years interval  Recalled consumption over previous year | Record linkage with national (Czech) or local mortality registers | Beer, wine, spirits | median follow-up 6.9 | Age, education, marital status, economic activity, asset score, subjective hardship score, smoking, physical activity, BMI, prevalent CVD and cancer, and  depressive symptoms | **Male**  Regular heavy (QPO>80 g >1 pw)VS Non-drinker HR  1.03 [0.76–1.39] VS 1.40 [1.13–1.74]  **Female**  Regular heavy (QPO>40 g >1 pw)VS Non-drinker HR:  0.38 [0.05–2.74] VS  1.47 [1.07–2.02] |
| Saito E et al (30) | 2018 | Japan | Japan Public Health Center-based Prospective | Cohort 1:40-69  Cohort 2: 40-59 | Male & Female | Male  48300  Female  54540 | Male  1203 heart disease deaths  Female  696 heart disease deaths | a self-report questionnaire  3 times in 5 years interval  Recalled current consumption status | Death certificates from the Ministry of Health, Labour and Welfare | Ethanol contained drinks: sake, shochu, awamori, whisky or brandy, wine, beer, | 18.2 on average | Age (years, continuous) and public health centre area, smoking status (never, former, <20 cigarettes=day, ≥20 cigarettes=day), BMI (<18.5, 18.5–<25, 25–<30, 30+), history of hypertension,  flushing response, history of diabetes, leisure-time sports or physical exercise (<almost daily, almost daily), intake of coffee and green tea (almost never, ≥1 cup=wk, and ≥1 cup=d), energy intake (continuous), intakes of fruits, vegetables, fish, meat, dairy products (continuous), and job status (employed or  unemployed), excluding deaths within 5 years of baseline. | **Male**  > 600g/week VS Non-drinkers HR :  0.87 (0.63–1.21),  p for non-liner trend <0.001,  p for linear trend in drinks <0.001  **Female**  > 600g/week VS Non-drinkers HR:  1.80 (0.56–5.76),  p for non-liner trend 0.001,  p for linear trend in drinks 0.009 |
| Ricci C et al (31) | 2020 | USA | NHANES | >18 | Male & Female | Male  16570  Female 18087 | Male  459 CVD deaths  Female  262 CVD deaths | Dietary interview and post-recall  2 times in 3-10 days  Recalled current consumption status | The US mortality registry | Alcohol | A median 7.8 | Ethnicity (Hispanic, black, white, others, or mixed), education (less or more than a high school degree), BMI (kg/m2; continuous), systolic and diastolic blood pressure (mm Hg;  continuous), smoking status (smoked ≥100 cigarettes in life),  energy intake (kcal/d), dietary fiber (g/d), and indicator variables for prevalent cardiovascular diseases (≥1 of the following: coronary artery disease, congestive heart failure, myocardial infarction, or stroke) and any primary cancer at baseline. | **Male**  ≥28 VS ≥0.1 and <14 VS<0.1g/d HR:  1.79 (1.04, 3.09) VS 1 VS1.81 (1.29, 2.53)  **Female**  ≥28 VS ≥0.1 and <14 VS <0.1g/d HR:  1.70 (0.83, 3.49)VS 1 VS 1.42 (0.91, 2.21) |
| Jankhotkaew J et al (32) | 2020 | Tailand | Electricity Generating Authority of Thailand (EGAT) | 35-54 | Total | 1961 | 64  CVD deaths | Questionnaire  5 times in 5, 12 years interval  Recalled consumption over previous year | The national vital registration | Alcohol | More than 30 years | Gender, age, marital status, education, monthly income, smoking status, physical activity, LDL, HDL and BMI to control for confounding effects. | Heavy drinkers VS Abstainers HR:  1.05 (0.10 to 11.12) |
| Armas Rojas NB et al (33) | 2021 | Cuba | NA | >30 | Total | 120623 | 5203  CVD deaths | Self-reported  Questionnaire  2 times in 8.6 years interval  Recalled current consumption status | National mortality records | Rum, beer | 17 (mean) | Age, sex, education, province, and smoking | > 3 bottles rum/week VS Non-drinker RR:  1.19 (1.05-1.34) VS 1.10 (1.06-1.15) |
| Zhang X et al (34) | 2021 | China | Kailuan Study | >18 | Total & Male | Total 83732  Male  65081 | 1594  CVD deaths | self-administered questionnaire  6 times in 2 years interval  Recalled consumption over previous year | Review of insurance and medical records | Beer, wine  or hard liquor | 10 mean | Age, sex, marriage (single, first marriage, other), education (elementary school or below, middle or high school, college or above), household income per capita (≤500, 501–1000, > 1000 Chinese yuan/month), smoking status (never, past, current), moderate/vigorous physical activity (< 1, 1–3, ≥4 times/week), sodium intake (< 6, 6–9.9, ≥10 g/day), body mass index (kg/m2), fasting blood glucose (mmol/L), anti- hypertension drugs (yes, no), anti-diabetes drugs (yes, no), and lipid-lowering drugs (yes, no). | **Total**  >750 HR alcohol (g/wk) VS 0:  1.46(0.78-2.27) VS 1.54 (1.24-1.91)  **Male**  >750g/wk VS 0 g/wk  HR:  0.93 (0.51, 1.70) VS 1,  P 0.01 |

**Table 4. Included study characteristics of investigating the association between Alcohol consumption and risk of cerebrovascular mortality in adults**

| **Author** | **Year** | **Country** | **Cohort name** | **Age at entry** | **Sex** | **Sample size** | **Total cases** | **Dietary assessment** | **Outcome assessment** | **Type of alcohol** | **Follow up years** | **Adjust factors** | **Results (The highest vs. the lowest intake category) HR or RR (95% CI)** |
| --- | --- | --- | --- | --- | --- | --- | --- | --- | --- | --- | --- | --- | --- |
| Streppel MT et al (26) | 2009 | Netherlands | Zutphen Study | Middle-aged | Male | 1373 | 139  Cerebrovascular deaths | Cross-check dietary history method  7 times in 5 years interval  Recalled consumption over previous month | Official death certificate | Beer, wine, spirits | 40 | Adjusted for former drinking, energy intake without alcohol, the number of cigarettes smoked, cigarette smoking duration, cigar or pipe smoking, intake of vegetables, fruit, fish, saturated fat and trans fatty acids, body mass index, prevalence of myocardial infarction, stroke, diabetes mellitus and cancer, and baseline socioeconomic status. | > 20g/day VS No drinker HR:  0.56 (0.25 to 1.25) |
| Jae Woong S et al (27) | 2010 | Korea | A Kangwha Cohort Study | > 55 | Male | 2600 | 245 cerebrovascular deaths | Alcohol consumption questionnaire  2 times in 9 years interval | Records of burial and death  certificates of eup and myeon offices, through the family’s confirmation of cause of death, or the national data on cause of death. | Consumed most soju and makkoli. | 20.8 | Age (year of recruitment), history of chronic disease, history of diabetes, smoking habits (never, former, 1–19 tobacco/day, and >20 tobacco/day), body mass index, hypertension status, occupation (agriculture, other), and education status, total alcohol consumption, excluding subjects who had been followed up <5 years. | Heavy (>12 Drinks) VS Non-drinker HR:  1.49 (0.61–3.64) |
| Pednekar MS et al (35) | 2012 | India | NA | >45 | Male | 35102 | 63  Cerebrovascular deaths | Interview  2 times in 5.5 years interval  Recalled consumption over previous year and present | Bombay Municipal Corporation(BMC) death registers | Country/desi liquor,  India made Foreignliquor, beer, sprits, toddy | Average 5.5 | Age, education, and tobacco use. | All types alcohol VS non-drinker HR:  1.83 (1.33-2.52) |
| Bobak M et al (29) | 2016 | Four Central and Eastern European countries | The Health, Alcohol and Psychosocial factors In Eastern Europe (HAPIEE) s | 45-69 | Male & Female | Male  15989  Female  18315 | Male  68 cerebrovascular death  Female  31 cerebrovascular death | Graduated frequency questionnaire  More than 2 times in 3.5 years interval  Recalled consumption over previous year | Record linkage with national (Czech) or local mortality registers | Beer, wine, spirits | median follow-up 6.9 | Age, education, marital status, economic activity, asset score, subjective hardship score, smoking, physical activity, BMI, prevalent CVD and cancer, and  depressive symptoms | **Male**  Regular heavy VS Non-drinker HR:  1.07(0.37-3.09) VS 1.23(0.55-2.75)  **Female**  Regular heavy VS Non-drinker HR:  6.38(0.64 -63.40) VS 0.94 (0.27-3.29) |
| Saito E et al (30) | 2018 | Japan | Japan Public Health Center-based Prospective | Cohort 1:40-69  Cohort 2: 40-59 | Male & Female | Male  48300  Female  54540 | Male  905 cerebrovascular disease deaths  Female  588 cerebrovascular disease deaths | a self-report questionnaire  3 times in 5 years interval  Recalled consumption current status | Death certificates from the Ministry of Health, Labour and Welfare | Ethanol contained drinks: sake, shochu, awamori, whisky or brandy, wine, beer, | 18.2 on average | Age (years, continuous) and public health centre area, smoking status (never, former, <20 cigarettes=day, ≥20 cigarettes=day), BMI (<18.5, 18.5–<25, 25–<30, 30+), history of hypertension,  flushing response, history of diabetes, leisure-time sports or physical exercise (<almost daily, almost daily), intake of coffee and green tea (almost never, ≥1 cup=wk, and ≥1 cup=d), energy intake (continuous), intakes of fruits, vegetables, fish, meat, dairy products (continuous), and job status (employed or  unemployed), excluding deaths within 5 years of baseline | **Male**  > 600g/week VS Non-drinkers HR:  1.36 (0.95–1.93)  p for non-liner trend <0.001,  p for linear trend in drinks 0.002  **Female**  > 600g/week VS Non-drinkers HR: 3.10 (1.32–7.32)  p for non-liner trend <0.001,  p for linear trend in drinks 0.020 |
| Ricci C et al (31) | 2020 | USA | NHANES | >18 | Male & Female | Male  16570  Female 18087 | Male  549  Cerebrovascular deaths  Female  337 cerebrovascular deaths | Dietary interview and post-recall  2 times in 3-10 days  Recalled consumption current status | The US mortality registry | Alcohol | A median 7.8 | Ethnicity (Hispanic, black, white, others, or mixed), education (less or more than a high school degree), BMI (kg/m2; continuous), systolic and diastolic blood pressure (mm Hg;  continuous), smoking status (smoked ≥100 cigarettes in life),  energy intake (kcal/d), dietary fiber (g/d), and indicator variables for prevalent cardiovascular diseases (≥1 of the following: coronary artery disease, congestive heart failure, myocardial infarction, or stroke) and any primary cancer at baseline. | **Male:**  ≥28g/d VS<0.1g/d HR:  1.96 (1.19, 3.22) VS 1.97 (1.44, 2.71)  **Female**  ≥28g/d VS<0.1g/d:  1.69 (0.86, 3.32) VS 1.43 (0.95, 2.15) |
| Armas Rojas NB et al (33) | 2021 | Cuba | NA | >30 | Total | 120623 | 1088  Cerebrovascular deaths | Self-reported  Questionnaire  2 times in 8.6 years interval  Recalled consumption current status | National mortality records | Rum, beer | 17 (mean) | Age, sex, education, province, and smoking | > 3 bottles rum/week VS Non-drinker RR:  1.13 (0.86-1.46) VS 0.78 (0.71-0.86) |

**Table 5. Included study characteristics of investigating the association between Alcohol consumption and risk of coronary heart disease (CHD) mortality in adults**

| **Author** | **Year** | **Country** | **Cohort name** | **Age at entry** | **Sex** | **Sample size** | **Total cases** | **Dietary assessment** | **Outcome assessment** | **Type of alcohol** | **Follow up years** | **Adjust factors** | **Results (The highest vs. the lowest intake category) HR or RR (95% CI)** |
| --- | --- | --- | --- | --- | --- | --- | --- | --- | --- | --- | --- | --- | --- |
| Shaper AG et al (24) | 2000 | UK | The British regional heart study | 45–64 | Male  with CHD | 655 | 175 CHD deaths | Alcohol  Questionnaire  2 times in 5 years interval | National Health Service registers | Alcohol | 12.8 | Age, smoking, BMI, social class, pre-existing diabetes, stroke, and regular medication | Moderate/heavy VS Teetotallers RR:  1.28 (0.83 to 1.96) VS 1.14 (0.60 to 2.18) |
| Beulens JWJ et al (25) | 2007 | USA | Health Professionals Follow-Up Study | 40-75 | Male  with hypertension | 11711 | 279  CHD deaths | food-frequency questionnaire  4 times in 4 years interval  Recalled consumption over previous year | Medical records or autopsy reports | Beer, white wine,  red wine, and liquor | 16 | Age; smoking; body mass index; physical activity; diabetes; hypercholesterolemia; family history of MI; aspirin use; lipid-lowering therapy; energy intake; and  energy-adjusted quintiles of saturated fat, trans fatty acids, sodium, potassium, magnesium, folate, vitamin E, -3 fatty acids, and dietary fiber. | > 50g/d VS 0 HR:  0.42 (0.17–1.04)  P trend 0.018 |
| Streppel MT et al (26) | 2009 | Netherlands | Zutphen Study | Middle-aged | Male | 1373 | 384  CHD deaths | Cross-check dietary history method  7 times in 5 years interval  Recalled consumption over previous month | Official death certificate | Beer, wine, spirits | 40 | Adjusted for former drinking, energy intake without alcohol, the  number of cigarettes smoked, cigarette smoking duration, cigar or pipe smoking,  intake of vegetables, fruit, fish, saturated fat and trans fatty acids, body mass index,  prevalence of myocardial infarction, stroke, diabetes mellitus and cancer, and baseline  socioeconomic status | > 20g/day VS No drinker HR:  0.77 (0.44 to 1.33) |
| Bobak M et al (29) | 2016 | Four Central and Eastern European countries | The Health, Alcohol and Psychosocial factors In Eastern Europe (HAPIEE) s | 45-69 | Male & Female | Male  15989  Female  18315 | Male  493 CHD deaths  Female  179 CHD deaths | Graduated frequency questionnaire  More than 2 times in 3.5 years interval  Recalled consumption over previous year | Record linkage with national (Czech) or local mortality registers | Beer, wine, spirits | median follow-up 6.9 | Age, education, marital status, economic activity, asset score, subjective hardship score, smoking, physical activity, BMI, prevalent CVD and cancer, and  depressive symptoms | **Male**  Regular heavy (QPO>80 g >1 pw)VS Non-drinker HR:  1.08 [0.73–1.59] VS 1.50 [1.13–1.99]  **Female**  Regular heavy (QPO>40 g >1 pw) VS Non-drinker  NA VS1.36 [0.86–2.16] |

**Table 6. Included study characteristics of investigating the association between Alcohol consumption and risk of ischemic heart disease (IHD) mortality in adults**

| **Author** | **Year** | **Country** | **Cohort name** | **Age at entry** | **Sex** | **Sample size** | **Total cases** | **Dietary assessment** | **Outcome assessment** | **Type of alcohol** | **Follow up years** | **Adjust factors** | **Results (The highest vs. the lowest intake category) HR or RR (95% CI)** |
| --- | --- | --- | --- | --- | --- | --- | --- | --- | --- | --- | --- | --- | --- |
| Jakovljević B et al (36) | 2004 | Serbia | NA | 30-60 | Total | 286 | 42  IHD deaths | Alcohol  Questionnaire  2 times in 20 years interval | Official death certificates | Beer, wine, spirits | 20 | Gender, smoking, body mass index, and blood pressure | Heavy VS Rarely or never RR:  2.463 (1.050-5.775)  P 0.038 |
| Pednekar MS et Al (35) | 2012 | India | NA | >45 | Male | 35102 | 146  IHD deaths | Interview  2 times in 5.5 years interval  Recalled consumption over previous year and present | Bombay Municipal Corporation(BMC) death registers | Country/desi liquor,  India made Foreignliquor, beer, sprits, toddy | Average 5.5 | Age, education, and tobacco use. | All types alcohol VS non-drinker HR:  0.90 (0.75-1.09) |
| Armas Rojas NB et al (33) | 2021 | Cuba | NA | >30 | Total | 120623 | 2548  IHD deaths | Self-reported  Questionnaire  2 times in 8.6 years interval  Recalled consumption current status | National mortality records | Rum, beer | 17 (mean) | Age, sex, education, province, and smoking | > 3 bottles rum/week VS Non-drinker RR:  1.16 (0.97-1.39) VS 1.18 (1.12-1.26) |

**Table 7. Included study characteristics of investigating the association between Tea consumption and risk of cardiovascular mortality in adults**

| **Author** | **Year** | **Country** | **Cohort**  **name** | **Age at entry** | **Sex** | **Sample size** | **Total cases** | **Dietary assessment** | **Outcome assessment** | **Type of tea** | **Follow up years** | **Adjusted factors** | **Results (The highest vs. the lowest intake category) Hazard Ratio (95% CI)** |
| --- | --- | --- | --- | --- | --- | --- | --- | --- | --- | --- | --- | --- | --- |
| Wang X et al | 2019 | China | The China-PAR project | NA | Total | 100902 | 1477 ASCVD deaths | Standardized  Questionnaire via interview  Collected at baseline and follow-up after 5 years  Recalled consumption over the previous year | Collected by interviewing study participants or their proxies, and checking hospital records and/or death certificates. | Green tea, black tea, and other types | 7.3 (medium) | Age, sex, region (north/south), area (rural/urban), cohort, education level (12 years or not), family history of ASCVD (yes or no), smoking (yes or no), drinking (yes or no), physical activity level (ideal or not), dietary factors (ideal or not for consumption of fresh vegetables and fruits, red meat, soy products, fish), body mass index, systolic blood pressure, fasting blood glucose, total cholesterol, high-density lipoprotein cholesterol | ASCVD mortality with habitual tea drinking:  3times/week HR VS Never or <3 times/week, HR  0.78 (0.69–0.88) |
| Ma L et al | 2023 | US | Nurses’ Health Study  Health Professionals Follow-Up Study | 30yr  40yr | Females with type 2 DM  Males with type 2 DM | 9252  3519 | 2397 CVD deaths | Food-frequency questionnaire  Collected at baseline and updated every two to four years | Reports by the next of kin or postal authorities or from searches of the National Death Index | NA | 18.5 | Analyses were adjusted for age (continuous), duration of diabetes mellitus (years), sex (men or women), white ethnicity (yes or no), physical activity (<3.0, 3.0-8.9,  9.0-17.9, 18.0-26.9, ≥27.0 metabolic equivalents-hours/week), smoking status (never, former, current 1-14 cigarettes/day, current ≥15 cigarettes/day), alcohol consumption (0, 0.1-4.9, 5.0-14.9, ≥15.0 g/day), menopausal status and post-menopausal hormone use (pre-menopause, post-menopause (never, former, or current hormone use), or missing; Nurses’ Health Study only), family history of type 2 diabetes (yes or no) or myocardial infarction (yes or no), intake of total energy (continuous), the modified Alternative Healthy Eating Index score (fourths), history of hypertension (yes or no) or hypercholesterolemia (yes or no), use of antihypertensive (yes or no) or lipid lowering drug (yes or no), aspirin use (yes or no), diabetes drug use (oral drug only, insulin use, or others), and change in body mass index before to after diabetes diagnosis. Individual beverage consumption was mutually adjusted. | CVD mortality in adults with tea >2 servings/day HRVS <1 serving/month HR:  0.89 (0.73 to 1.09), P 0.03 |

**Table 8. Included study characteristics of investigating the association between Sugar-sweetened beverages (SSBs) and risk of cardiovascular mortality in adults**

| **Author** | **Year** | **Country** | **Cohort name** | **Age at entry** | **Sex** | **Sample size** | **Total cases** | **Dietary assessment** | **Outcome assessment** | **Type of SSB and ASB** | **Follow up years** | **Adjusted factors** | **Results (The highest vs. the lowest intake category) Hazard Ratio (95% CI)** |
| --- | --- | --- | --- | --- | --- | --- | --- | --- | --- | --- | --- | --- | --- |
| Vasanti S. et.al | 2019 | USA | Health Professional’s Follow-up study (HPFS)  The Nurses’ Health study(NHS) | Men aged 40 to 75  Women 30 to 55 | Total | 80 647 women  37 716 men | 7896 CVD deaths | Food-frequency questionnaire  HPFS; 7 times in 4 years interval  NHS: 8 times in 4 years interval  Recalled consumption over the previous year | State vital statistics records and the National Death Index or by reports from next of kin or the postal authorities | Caffeinated colas, caffeine-free colas, other  (ie, noncola) carbonated sugar-sweetened beverages, and noncarbonated sugar-sweetened beverages (fruit punches,  lemonades, or other fruit drinks).  ASBs were defined as caffeinated, caffeinefree, and noncarbonated low-calorie or diet beverages | 28 /HPFS  34/NHS | Age, smoking, alcohol intake, postmenopausal hormone use (NHS), physical activity, family history of diabetes, family  history of myocardial infarction, family history of cancer, multivitamin use, ethnicity, and aspirin use,  baseline history of hypertension and hypercholesterolemia; intake of whole grains, fruit, vegetables, red and  processed meat; total energy; and body mass index. | CVD mortality with SSB (two pooled data  ≥ 2/d VS ≤1/mo  1.31(1.15, 1.50),  p <0.0001 |
| Ma L et al | 2023 | US | Nurses’ Health Study  Health Professionals Follow-Up Study | 30yr  40yr | Females with type 2 DM  Males with type 2 DM | 9252  3519 | 2397 CVD deaths | Food-frequency questionnaire  Collected at baseline and updated every two to four years | Reports by the next of kin or postal authorities or from searches of the National Death Index | SSBs included caffeinated colas, caffeine-free colas, other carbonated SSBs, and non-carbonated SSBs (fruit punches, lemonades, or other fruit drinks). ASBs included low calorie cola with caffeine, low calorie caffeine-free cola, and other low calorie beverages | 18.5 | Analyses were adjusted for age (continuous), duration of diabetes mellitus (years), sex (men or women), white ethnicity (yes or no), physical activity (<3.0, 3.0-8.9,  9.0-17.9, 18.0-26.9, ≥27.0 metabolic equivalents-hours/week), smoking status (never, former, current 1-14 cigarettes/day, current ≥15 cigarettes/day), alcohol consumption (0, 0.1-4.9, 5.0-14.9, ≥15.0 g/day), menopausal status and post-menopausal hormone use (pre-menopause, post-menopause (never, former, or current hormone use), or missing; Nurses’ Health Study only), family history of type 2 diabetes (yes or no) or myocardial infarction (yes or no), intake of total energy (continuous), the modified Alternative Healthy Eating Index score (fourths), history of hypertension (yes or no) or hypercholesterolemia (yes or no), use of antihypertensive (yes or no) or lipid lowering drug (yes or no), aspirin use (yes or no), diabetes drug use (oral drug only, insulin use, or others), and change in body mass index before to after diabetes diagnosis. Individual beverage consumption was mutually adjusted. | CVD mortality with SSBs >1 serving/day HR VS <1 serving/month HR:  1.29 (1.02 to 1.63)  P 0.003 |

**Table 9. Included study characteristics of investigating the association between artificially sweetened beverage (ASBs) and risk of cardiovascular mortality in adults**

| **Author** | **Year** | **Country** | **Cohort name** | **Age at entry** | **Sex** | **Sample size** | **Total cases** | **Dietary assessment** | **Outcome assessment** | **Type of SSB and ASB** | **Follow up years** | **Adjusted factors** | **Results (The highest vs. the lowest intake category) Hazard Ratio (95% CI)** |
| --- | --- | --- | --- | --- | --- | --- | --- | --- | --- | --- | --- | --- | --- |
| Vasanti S. et.al | 2019 | USA | Health Professional’s Follow-up study (HPFS)  The Nurses’ Health study(NHS) | Men aged 40 to 75  Women 30 to 55 | Total | 80 647 women  37 716 men | 7896 CVD deaths | Food-frequency questionnaire  HPFS; 7 times in 4 years interval  NHS: 8 times in 4 years interval  Recalled consumption over the previous year | State vital statistics records and the National Death Index or by reports from next of kin or the postal authorities | Caffeinated colas, caffeine-free colas, other  (ie, noncola) carbonated sugar-sweetened beverages, and noncarbonated sugar-sweetened beverages (fruit punches,  lemonades, or other fruit drinks).  ASBs were defined as caffeinated, caffeinefree, and noncarbonated low-calorie or diet beverages | 28 /HPFS  34/NHS | Age, smoking, alcohol intake, postmenopausal hormone use (NHS), physical activity, family history of diabetes, family  history of myocardial infarction, family history of cancer, multivitamin use, ethnicity, and aspirin use,  baseline history of hypertension and hypercholesterolemia; intake of whole grains, fruit, vegetables, red and  processed meat; total energy; and body mass index. | CVD mortality with ASB  ≥ 2/d VS ≤1/w (two pooled data)  1.13 (1.02, 1.25)  P 0.02 |
| Ma L et al | 2023 | US | Nurses’ Health Study  Health Professionals Follow-Up Study | 30yr  40yr | Females with type 2 DM  Males with type 2 DM | 9252  3519 | 2397 CVD deaths | Food-frequency questionnaire  Collected at baseline and updated every two to four years | Reports by the next of kin or postal authorities or from searches of the National Death Index | SSBs included caffeinated colas, caffeine-free colas, other carbonated SSBs, and non-carbonated SSBs (fruit punches, lemonades, or other fruit drinks). ASBs included low calorie cola with caffeine, low calorie caffeine-free cola, and other low calorie beverages | 18.5 | Analyses were adjusted for age (continuous), duration of diabetes mellitus (years), sex (men or women), white ethnicity (yes or no), physical activity (<3.0, 3.0-8.9,  9.0-17.9, 18.0-26.9, ≥27.0 metabolic equivalents-hours/week), smoking status (never, former, current 1-14 cigarettes/day, current ≥15 cigarettes/day), alcohol consumption (0, 0.1-4.9, 5.0-14.9, ≥15.0 g/day), menopausal status and post-menopausal hormone use (pre-menopause, post-menopause (never, former, or current hormone use), or missing; Nurses’ Health Study only), family history of type 2 diabetes (yes or no) or myocardial infarction (yes or no), intake of total energy (continuous), the modified Alternative Healthy Eating Index score (fourths), history of hypertension (yes or no) or hypercholesterolemia (yes or no), use of antihypertensive (yes or no) or lipid lowering drug (yes or no), aspirin use (yes or no), diabetes drug use (oral drug only, insulin use, or others), and change in body mass index before to after diabetes diagnosis. Individual beverage consumption was mutually adjusted. | CVD mortality with ASBs >2 serving/day HR VS <1 serving/month HR:  0.93 (0.75 to 1.14)  P 0.94 |

**Supplementary Table 10. Quality assessment**

| **Author** | **Representativeness of the exposed cohort** | **Selection of the non exposed cohort** | **Ascertainment of exposure** | **Demonstration that outcome of interest was not present at start of study** | **The comparability of the groups** | **Assessment of outcome** | **Was follow-up long enough for outcomes to occur** | **Adequacy of follow-up of cohorts** | **Quality assessment** |
| --- | --- | --- | --- | --- | --- | --- | --- | --- | --- |
| **Tea** | | | | | | | | | |
| Xinyan Wang et al | b) | a) | b) | a) | a) | b) | b) | a) | Good quality |
| **Sugar-sweetened beverages** | | | | | | | | | |
| Vasanti S. et.al | c) | a) | c) | a) | a) | b) | a) | a) | Good quality |
| **Artificially sweetened beverage** | | | | | | | | | |
| Ma L et al c) a) b) a) a) and b) b) a) a) Good quality | | | | | | | | | |
| **Coffee** | | | | | | | | | |
| Jazbec Anamarijaet al | c) | a) | b) | a) | c) | b) | a) | a) | Good quality |
| James A Greenberg et.al | c) | a) | b) | a) | a) | b) | b) | a) | Good quality |
| Esther Lopez-Garcia et al | b) | a) | c) | a) | a) | b) | a) | a) | Good quality |
| WEILI ZHANG et al | c) | a) | c) | a) | a) | e) | a) | a) | Fair quality |
| **Alcohol** | | | | | | | | | |
| A G Shaper et. Al | c) | a) | b) | a) | a) | b) | a) | a) | Good quality |
| Branko Jakovljevic et al | c) | c) | c) | a) | a) | b) | a) | c) | Poor quality |
| Joline W.J. et al | c) | a) | c) | a) | a) | b) | a) | a) | Good quality |
| M T Streppel et al | c) | a) | c) | a) | a) | b) | a) | b) | Fair quality |
| Jae Woong Sull et al | c) | a) | b) | a) | a) | b) | a) | a) | Good quality |
| Gundula Behrens et al | c) | a) | c) | a) | a) | b) | a) | a) | Fair quality |
| Mangesh S. Pednekar et. Al | c) | a) | b) | a) | a) | b) | b) | a) | Fair quality |
| Martin Bobak et.al | c) | a) | c) | a) | a) | b) | b) | a) | Fair quality |
| Eiko Saito et.al | b) | a) | c) | a) | a) | b) | a) | a) | Good quality |
| Cristian Ricci et.al | c) | a) | b) | a) | c) | b) | b) | a) | Fair quality |
| Jintana Jankhotkaew et al | b) | a) | c) | a) | a) and b) | b) | a) | a) | Good quality |
| Nurys B. Armas Rojas, MD et al | b) | a) | c) | a) | a) and b) | b) | a) | b) | Good quality |
| Xinyuan Zhang et al | b) | a) | c) | a) | a) and b) | b) | a) | a) | Good quality |

**Supplementary Figure 1. Funnel plot showing study precision against the hazard ratio with 95% CIs for coffee consumption and cardiovascular mortality among both sexes**

**
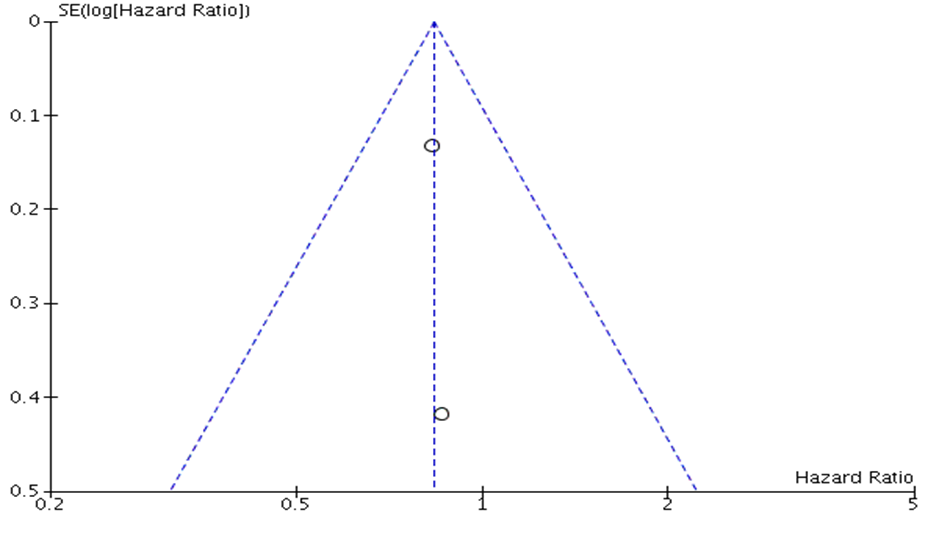
**

**Supplementary Figure 2. Funnel plot showing study precision against the hazard ratio with 95% CIs for coffee consumption and cardiovascular mortality among males**


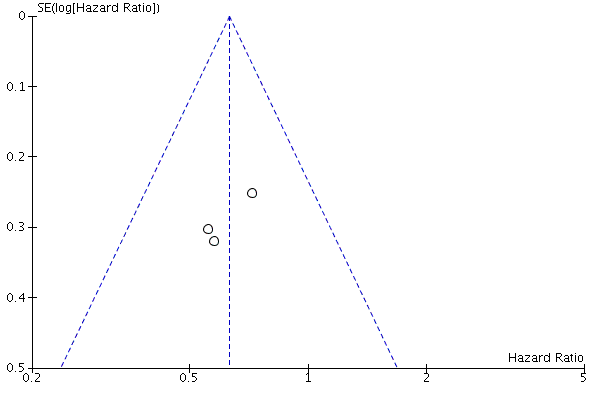


**Supplementary Figure 3. Funnel plot showing study precision against the hazard ratio with 95% CIs for coffee consumption and cardiovascular mortality among females**


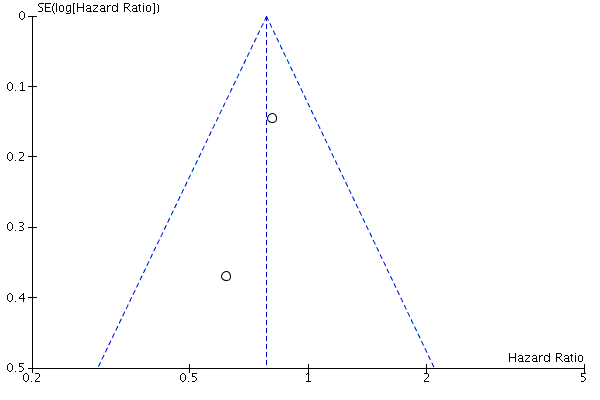
**Supplementary Figure 4. Funnel plot showing study precision against the hazard ratio with 95% CIs for alcohol consumption and overall cardiovascular mortality among adults.**


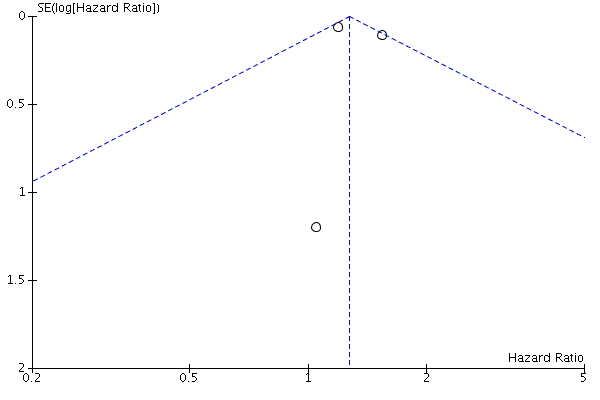


**Supplementary Figure 5. Funnel plot showing study precision against the hazard ratio with 95% CIs for alcohol consumption and overall cardiovascular mortality among males.**


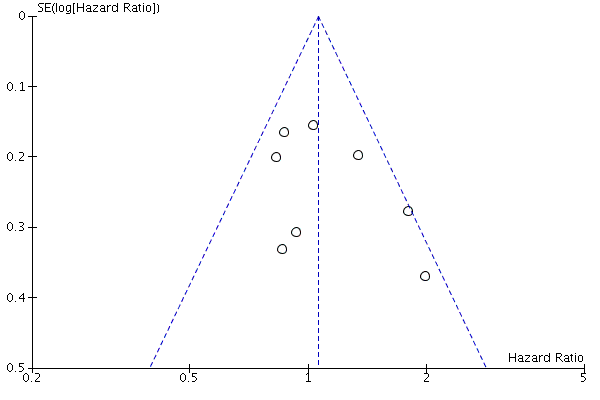


**Supplementary Figure 6. Funnel plot showing study precision against the hazard ratio with 95% CIs for alcohol consumption and overall cardiovascular mortality among females**


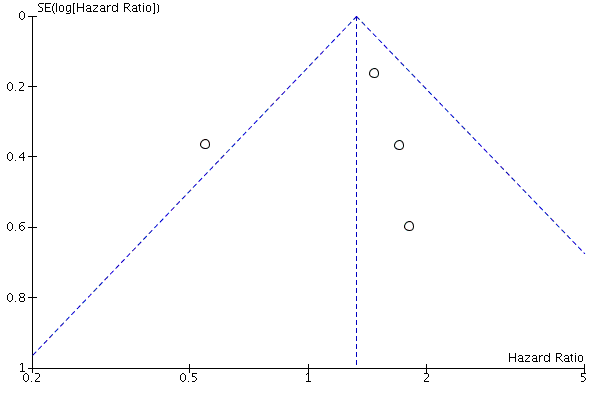


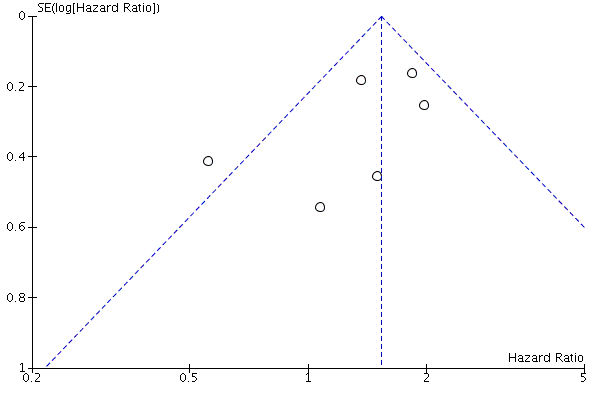
**Supplementary Figure 7. Funnel plot showing study precision against the hazard ratio with 95% CIs for alcohol consumption and stroke mortality among males**

**Supplementary Figure 8. Funnel plot showing study precision against the hazard ratio with 95% CIs for alcohol consumption and stroke mortality among females**


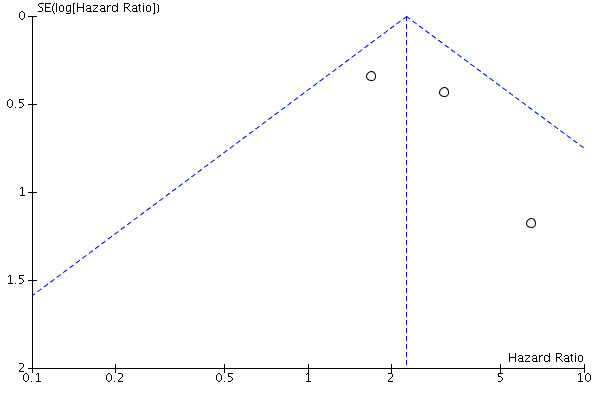


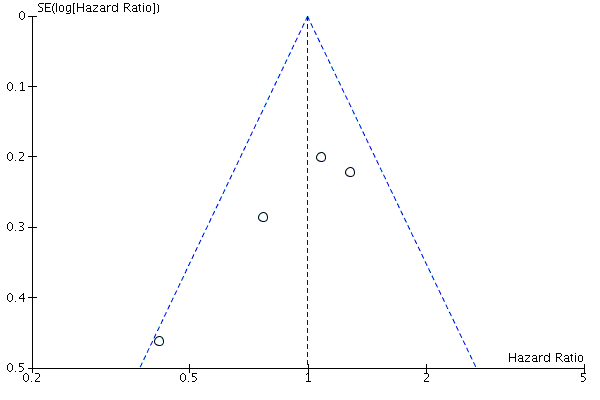
**Supplementary Figure 9. Funnel plot showing study precision against the hazard ratio with 95% CIs for alcohol consumption and coronary heart disease mortality among males**

**Supplementary Figure 10. Funnel plot showing study precision against the hazard ratio with 95% CIs for alcohol consumption and ischemic heart disease mortality among adults**


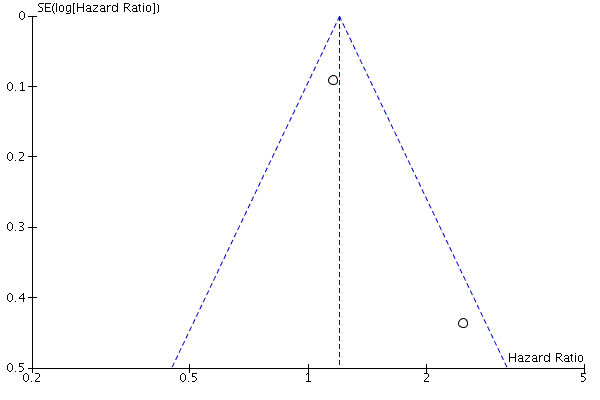


**
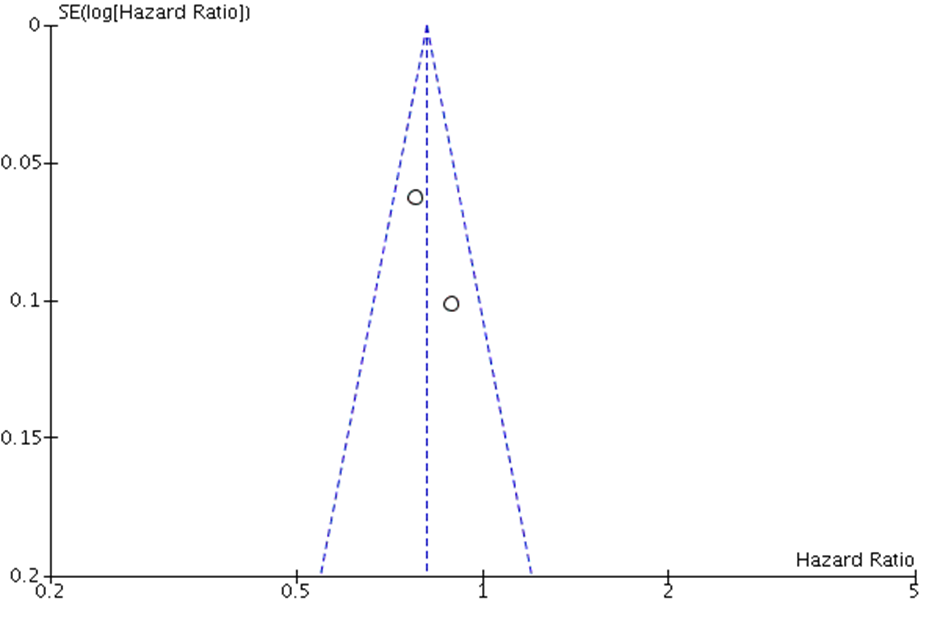
Supplementary Figure 11. Funnel plot showing study precision against the hazard ratio with 95% CIs for tea consumption and overall cardiovascular mortality among both sexes**

**Supplementary Figure 12. Funnel plot showing study precision against the hazard ratio with 95% CIs for sugar-sweetened beverage consumption and overall cardiovascular mortality among both sexes**

**
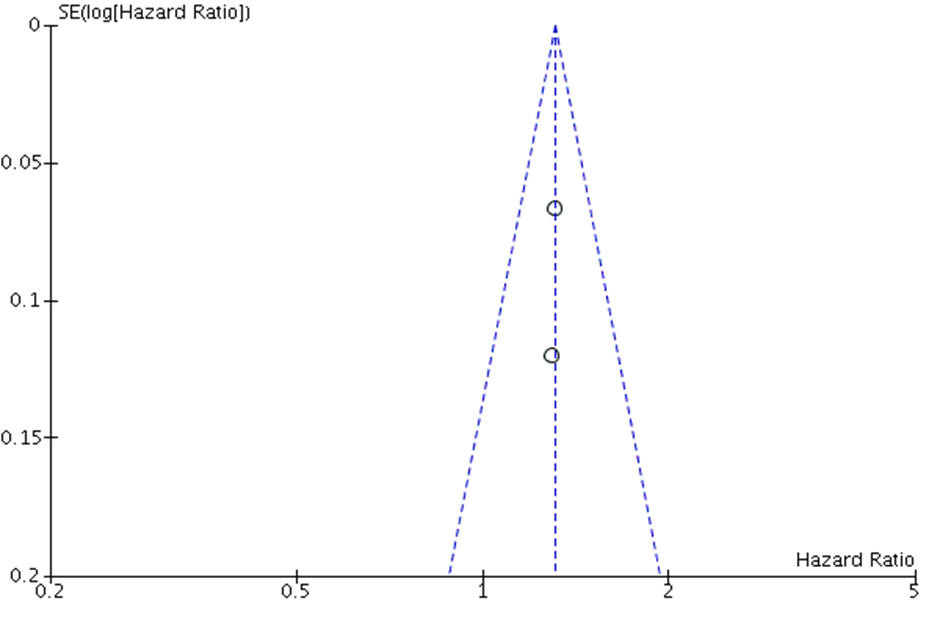
**

**Supplementary Figure 13. Funnel plot showing study precision against the hazard ratio with 95% CIs for artificially sweetened beverage consumption and overall cardiovascular mortality among both sexes**

**
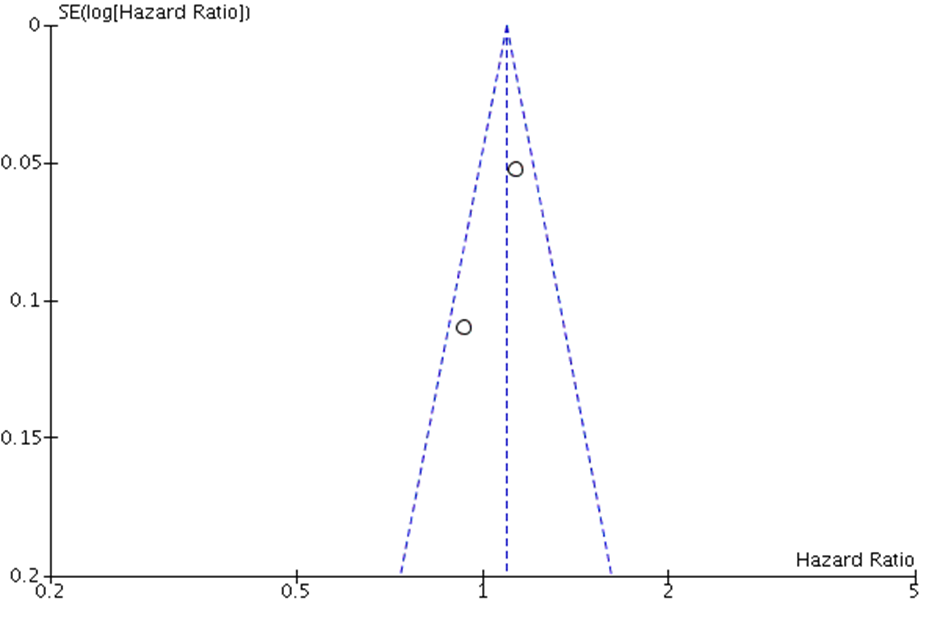
**
